# Cystathionine gamma-lyase (CTH) inhibition attenuates glioblastoma formation

**DOI:** 10.1016/j.redox.2023.102773

**Published:** 2023-06-05

**Authors:** Maria Peleli, Ivi Antoniadou, Dorival Mendes Rodrigues-Junior, Odysseia Savvoulidou, Laia Caja, Antonia Katsouda, Daniel F.J. Ketelhuth, Jane Stubbe, Kirsten Madsen, Aristidis Moustakas, Andreas Papapetropoulos

**Affiliations:** a Department of Medical Biochemistry and Microbiology, Science for Life Laboratory, Uppsala University, Box 582, SE-751 23, Uppsala, Sweden; b Clinical, Experimental Surgery and Translational Research Center, Biomedical Research Foundation of the Academy of Athens, Athens, Greece; c Laboratory of Pharmacology, Department of Pharmacy, National and Kapodistrian University of Athens, Athens, Greece; d Department of Cardiovascular and Renal Research, Institute of Molecular Medicine, University of Southern Denmark, J. B. Winslowsvej 21, 3, 5000, Odense C, Denmark; e Division of Cardiovascular Medicine, Center for Molecular Medicine, Department of Medicine, Karolinska Institute, Karolinska University Hospital, Stockholm, Sweden; f Department of Pathology, Odense University Hospital, J.B Winslowsvej 15, 5000, Odense C, Denmark

**Keywords:** Cystathionine gamma-lyase (CTH), Glioblastoma stem cells (GSC), Brain blood vessels, Sex determining region Y-Box 2 (SOX2)

## Abstract

**Purpose:**

Glioblastoma (GBM) is the most common type of adult brain tumor with extremely poor survival. Cystathionine-gamma lyase (CTH) is one of the main Hydrogen Sulfide (H_2_S) producing enzymes and its expression contributes to tumorigenesis and angiogenesis but its role in glioblastoma development remains poorly understood.

**Methods:**

and Principal Results: An established allogenic immunocompetent *in vivo* GBM model was used in C57BL/6J WT and *CTH* KO mice where the tumor volume and tumor microvessel density were blindly measured by stereological analysis. Tumor macrophage and stemness markers were measured by blinded immunohistochemistry. Mouse and human GBM cell lines were used for cell-based analyses. In human gliomas, the CTH expression was analyzed by bioinformatic analysis on different databases.

*In vivo*, the genetic ablation of CTH in the host led to a significant reduction of the tumor volume and the protumorigenic and stemness transcription factor sex determining region Y-box 2 (SOX2). The tumor microvessel density (indicative of angiogenesis) and the expression levels of peritumoral macrophages showed no significant changes between the two genotypes. Bioinformatic analysis in human glioma tumors revealed that higher CTH expression is positively correlated to SOX2 expression and associated with worse overall survival in all grades of gliomas. Patients not responding to temozolomide have also higher CTH expression. In mouse or human GBM cells, pharmacological inhibition (PAG) or CTH knockdown (siRNA) attenuates GBM cell proliferation, migration and stem cell formation frequency.

**Major Conclusions:**

Inhibition of CTH could be a new promising target against glioblastoma formation.

## Highlights

• CTH inhibition in the tumor host attenuates glioblastoma.
• CTH is upregulated in human gliomas and associated with worse overall survival.
• CTH inhibition lowers GBM cell proliferation, migration and stem cell formation.

## Graphical abstract

Overview of the proposed mechanisms by which CTH might regulate glioblastoma formation. Pharmacological (PAG) inhibition of CTH on human glioblastoma cells results to lower Glioblastoma Stem Cell (GSC) formation and lower mRNA expression of stem cell markers (i.e, PROM1, NOX4). Bioinformatic analysis reveals also higher CTH expression in human GBM, which is associated with worse overall survival, non-response to temozolomide (TMZ) and higher expression of the GSC marker SOX2. Inhibition of CTH in mouse GBM cells with a brain-permeable pharmacological inhibitor (PAG) or knockdown with siRNA (siCTH) results in lower GBM cell proliferation and migration by an H_2_S-dependent mechanism, since the effects of CTH inhibition are attenuated by the H_2_S donor, NaSH. CTH expression in the tumor stroma/microenvironment plays a crucial role for glioblastoma formation and triggers the expression of the GSC marker SOX2 without affecting the tumoral vascular network. *CTH* KO mice with GBM have significantly lower tumor volume, tumoral SOX2 expression and no alteration in the levels of tumoral vascular density compared to WT mice with GBM. The figure was created by using the Biorender software https://biorender.com/.

**Figure:**
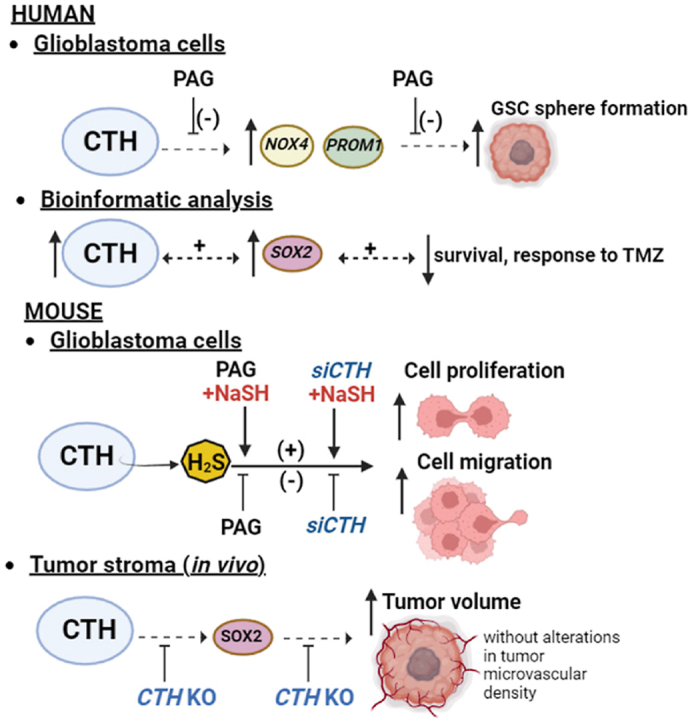

## Abbreviations

**bFGF**: basic Fibroblast Growth Factor
**BSA**: Bovine Serum Albumin
**CD133 or PROM1**: Cluster of Differentiation antigen 133 or Prominin-1
**CD31 or PECAM1**: Cluster of Differentiation antigen 31 or Platelet And Endothelial Cell Adhesion Molecule 1
**CD34**: Cluster of Differentiation antigen 34 (Sialomucin)
**CD44**: Cluster of Differentiation antigen 44 (homing-associated cell adhesion molecule)
**cDNA**: complementary Deoxyribonucleic acid
**CLDN5**: Claudin-5
**CGGA**: Chinese Glioblastoma Genome Atlas
**CTH**: Cystathionine-gamma lyase
**CTH KO**: cystathionine-gamma lyase knockout
**DMSO**: Dimethyl sulfoxide
**EGF**: Epidermal Growth Factor
**ELDA**: Extreme Limiting Dilution Analysis
**GBM**: Glioblastoma
**GSC**: Glioblastoma Stem Cells
**GSH**: Glutathione
**GTEx**: Genotype-Tissue Expression
**H&E**: Hematoxylin&Eosin
**H**_**2**_**O**_**2**_: Hydrogen Peroxide
**H**_**2**_**S**: Hydrogen Sulfide
**IDH-1**: Isocitrate dehydrogenase-1
**IHC**: Immunohistochemistry
**MAC-2**: Galectin-3
**NADPH**: Nicotinamide Adenine Dinucleotide Phosphate
**NES**: Nestin
**NOX4**: NADPH oxidase 4
**OS**: Overall Survival
**PAG**: l-propargylglycine
**PBS**: Phosphate Buffered Saline
**PFA**: Paraformaldehyde
**qPCR**: quantitative Polymerase Chain Reaction
**RFU**: Relative Fluorescence Unit
**RNA**: Ribonucleic acid
**RNA-seq**: RNA sequencing
**ROC**: Receiver Operating Characteristic
**ROS**: Reactive Oxygen Species
**SOX2, 4**: Sex determining region Y-box-2, -4
**TAMs**: Tumor Associated Macrophages
**TCGA**: The Cancer Genome Atlas
**TGF-β1**: Transforming growth factor-beta 1
**WT**: wildtype

## 1 Introduction

Glioblastoma (GBM) is the most prevalent and aggressive primary brain tumor in adults with a median overall survival of 15 months. GBM tumors are characterized by high invasiveness, inflammation, increased microvascular density and endothelial cell proliferation [1]. The main current therapeutic approaches against GBM is surgical resection of the tumor followed by chemotherapy (mostly temozolomide) and/or radiotherapy [2]. More recently, and based on the importance of the tumor vasculature for the progression of glioblastoma formation [3], some anti-angiogenic treatments have also been used such as bevacizumab, which however showed no increase in overall survival (OS) but improved the progression-free survival [2,4]. Despite the advances in the current diagnostics and therapeutics GBM remains the most deadly and aggressive type of brain tumor with a 5-year survival of less than 5% [5]. Therefore, identifying new molecular players involved in glioblastoma formation is imperative in order to improve the odds of survival and the quality of life of these patients.

Cystathionine-gamma lyase (CTH) is one of the main enzymes involved in the production of the gasotransmitter hydrogen sulfide (H_2_S) in the body by using cystathionine and cysteine as substrates [6]. This enzyme is part of the so-called transsulfuration pathway that contributes significantly to the pools of the important antioxidant molecule glutathione (GSH) in the brain [7]. CTH-derived H_2_S has been shown to be an important endogenous inducer of angiogenesis in the whole body, including the brain [8,9]. Moreover, CTH can promote tumorigenesis via supporting angiogenesis mechanisms in other cancer types such as breast or prostate cancer [10,11]. Whether CTH is able of supporting angiogenesis-related mechanisms during glioblastoma formation remains unknown. A recent study analyzing the impact of antioxidant GSH in specific subtypes of gliomas, indicated the importance of CTH as precursor enzyme for high GSH synthesis in astrocytoma cells [12]. This study relied on xenografts in immunocompromised mice and did not examine the importance of the tumoral vascular network or more generally, the role of CTH in the tumor stroma cells of glioblastoma.

Investigating the role of CTH in the tumoral stroma alone, using immunocompetent mice, and comparing it with the role of CTH in isolated GBM cells, would therefore give us a deeper understanding about the role of this enzyme in GBM. Such an approach would also allow us to understand if targeting CTH in the tumoral stroma, the GBM cells or both could have any therapeutic potential.

Our results show that CTH expressed in the tumor microenvironment supports glioblastoma formation without affecting the tumoral microvascular density. We also show that CTH is highly expressed in human GBM tumors and correlates with worse overall survival and non-response to temozolomide but not with anti-angiogenesis treatments. Finally, CTH knockdown (siRNA) or pharmacological inhibition of CTH with a brain permeable drug on isolated GBM cells results in lower GBM cell proliferation, migration and stem cell formation.

Therefore, the use of CTH-specific blood brain barrier permeable pharmacological or molecular inhibitors could have a therapeutic potential against GBM.

## 2 Materials and Methods

### 2.1 Cell culture

The patient-derived glioblastoma U3031MG, U3017MG, U3034MG cells were acquired and authenticated by the Human Glioblastoma Cell Culture resource (www.hgcc.se) at the Dept. of Immunology, Genetics and Pathology, Uppsala University, Uppsala, Sweden, using whole genome sequencing [13]. The mouse GL261 GBM cells were a gift by Prof. Anna Dimberg (Uppsala University, Sweden). The cells were cultured as previously described [14,15], details of the protocols are given in the **Supplement**.

### 2.2 Cell culture treatments

Cells were treated under the following conditions: I) ‘Control’ were treated with ‘vehicle’ (0.1% bovine serum albumin (BSA)/4 mM HCl solvent for Transforming growth factor-beta 1 (TGF-β1) and/or 0.1% DMSO (solvent for PAG), II) ‘PAG’ were treated with the CTH pharmacological inhibitor PAG (l-propargylglycine, Sigma, 600 μΜ, tock in DMSO, final DMSO concentration in the cell culture 0.1%) III) ‘TGF-β’ were treated with recombinant mouse TGF-β1 (PeproTech EC Ltd, London, UK, 5 ng/ml final concentration, stock solution 5 μg/ml in 0.1% BSA/4 mM HCl). PAG was always administered 30 min before the addition of TGF-β1 or the vehicle in case of ‘control’. All treatments were performed on full (non-starvation) media.

### 2.3 siRNA transfection and treatment protocol

All reagents for the siRNA transfection experiments in mouse GL261 glioblastoma cells were purchased by Thermofischer (Invitrogen) as follows: siRNA for CTH purchased under the commercial name ‘CTH siRNA (mouse), siRNA ID 173374, size 5 nmol, Cat. No. AM16708’, siRNA for Control (scrambled siRNA) under the commercial name ‘Silencer™ Select Negative Control No. 1 siRNA, size 5 nmol, Cat. No: 4390843’, Opti-MEM® I Reduced Serum Medium, (Cat. No. 31985-062) and Lipofectamine RNAiMAX (Cat. No. 13778030). The protocol of reverse siRNA transfection (transfection at the moment of cell plating) by using Lipofectamine in Opti-MEM® I Reduced Serum Medium was performed according to the manufacturer's (Invitrogen) instructions. The cells were plated in media supplemented with normal FBS (10%) but without antibiotics. After performing some trial experiments, we concluded that the optimal final siRNA transfection concentration that led to a stable (∼70%) downregulation of CTH was 30 nM and this concentration was used for all the subsequent cell proliferation and cell migration assays.

### 2.4 Sphere formation assay (ELDA assay)

Briefly, the human GBM U3017MG and U3034MG cells were seeded in Corning, Costar, Ultra-Low attachment 96-well plates (Corning Incorporated, Corning, NY, USA) performing a serial dilution from 100 to 3 cells in 6 replicates for each dilution and experimental condition in a volume of 100 μl N2B27 medium. Cells were incubated for 9 days with vehicle (DMSO 0.1%) or PAG (600 μΜ); then, the wells with neurospheres >50 μm were scored as positive and the actual number of spheres in each well was counted. The neurospheres were visualized using a phase-contrast Axiovert 40 CFL microscope (Carl-Zeiss, Oberkochen, Germany). The data were processed by the R package Extreme Limiting Dilution Analysis (ELDA) program [16].

### 2.5 Scratch assay

Scratch assays were performed as previously described with some slight modifications [17]. Scratch assays were performed by performing scratches in a form of a ‘cross’ (one horizontal and one vertical scratch with a sterile 200 μl tip) as previously described [17]. The cells were visualized and photographed with the ImageXpress® Pico Automated Cell Imaging System at 0, 6 and 24 h after scratching. At 24 h the wounds were completely closed and therefore the comparison of the 0 to 6 h-time points was used for the analysis. The % of scratching area and the number of migrating cells was counted for each time point on Image-J by two independent researchers and at the end the % of wound closure and % of cell migration respectively was calculated.

### 2.6 MTS cell viability and cell proliferation assays

The % of cell viability and cell proliferation was monitored in a spectrophotometer following the manufacturer’s protocol (Promega, Biotech AB, Nacka, Sweden). For cell viability, the cells were plated in 96-well plates at confluent concentrations (10^4^ cells/well for mouse GBM cells and 3x10^3^ cells/well for human GBM cells) whereas for the cell proliferation assays the mouse glioblastoma cells were plated in sub-confluent concentrations (2.5 × 10^3^ cells/well). After allowing 1 day for cell attachment, the cell treatments were performed as explained above and the cell viability was assessed 48 h after the treatments were added whereas the cell proliferation 24 h after. Further details of the protocol are given in the **Supplement**.

### 2.7 Cell proliferation by manual cell counting and trypan blue staining

Mouse GL261 glioblastoma cells were plated in 6-well plates in sub-confluent concentrations (3.8 × 10^4^ cells/well) and were treated the following day for 24 h. The cells in each well were then harvested and counted as follows: removal of medium, washing 2 × with DPBS, trypsinization (80 μl/well) and addition of 220 μl of full (10% FBS) media (final volume 300 μl), transfer of cells to new Eppendorf tubes on ice and addition of filtered trypan blue dye (10% final concentration), cell counting on a hematocytometer and calculation of the total alive and dead cell number. The level of cell death did not differ significantly among the different tested experimental conditions and was on average 8.54% ± 0.98. The cell proliferation was calculated based on the total number of alive cells for each experimental condition and at the end it was expressed as % of the ‘Control’ treated cells. The cell counting was performed by two independent researchers to minimize bias and the average of the two was used for the final analysis.

### 2.8 RNA extraction, cDNA synthesis and Real Time PCR

Real Time PCR was performed on total cellular extracted RNA and reverse transcribed cDNA with a SYBR green-based detection method. The details of the protocol and the primer sequences are given in the Supplemental section of Materials and Methods and in the Supplemental Table 1.

### 2.9 Orthotopic GL261 mouse glioma model

The orthotopic GL261 glioblastoma model was performed with some slight modifications as described in the Supplement [15].

### 2.10 Stereology analysis of tumor volume, % of tumor area, tumor microvessel density

Blinded stereologic measurements were done as previously described [[18], [19], [20]] with some appropriate modifications for the brain tissue. The details of the protocol are given in the Supplement.

### 2.11 Immunohistochemistry (IHC) analysis

Slices were stained with antibodies specific for SOX2 (Millipore, Cat No AB5603), MAC-2 (Cedarlane Laboratories, Burlington, Canada, Cat No CL8942AP) and CD34 (Abcam, Cat No ab8158) following standard IHC protocols as described previously [[21], [22], [23]]. Details of the protocol are given in the Supplement.

### 2.12 Bioinformatic analysis

The Cancer Genome Atlas (TCGA) and Genotype-Tissue Expression (GTEx) datasets were used to assess the expression of CTH in GBM compared to normal brain tissue using GEPIA2 (http://gepia2.cancer-pku.cn) [24]. The cBioPortal for Cancer Genomics [25] was used to retrieve RNA-seq data from GBM patients and gene coexpression analyses of CTH with other genes of interest. Correlation of gene expression with treatment response to temozolomide and angiogenesis inhibitors in GBM patients was conducted using the database from ROC Plotter (https://www.rocplot.org/gbm) as previously described [26,27]. GBM patients were categorized as responders or non-responders, according to the survival status at 16 months post-surgery, and gene expression was compared with the Mann-Whitney *U* test. Additionally, the Chinese Glioma Genoma Atlas (CGGA) was used to retrieve mRNA microarray and RNA-seq data of GBM patients and generate survival Kaplan-Meier plots as previously described [28].

### 2.13 Statistical analysis

The cell culture, *in vivo* stereology and immunohistochemistry, data were analyzed by using Excel and GraphPad Prism 9.0 software. Unpaired Student’s t-test was used for comparison between two groups, comparisons between three groups were performed by ordinary one-way ANOVA followed by Bonferroni’s multiple comparisons test, and comparisons between three groups over time were performed by two-way ANOVA followed by Bonferroni’s multiple comparisons test. All statistical tests were two-sided, and P values smaller than 0.05 were considered statistically significant. SEM was indicated as an error bar in all the figures.

## 3 Results

### 3.1 Genetic ablation of CTH in the mouse host is sufficient for attenuating glioblastoma formation

**Fig. 1:**
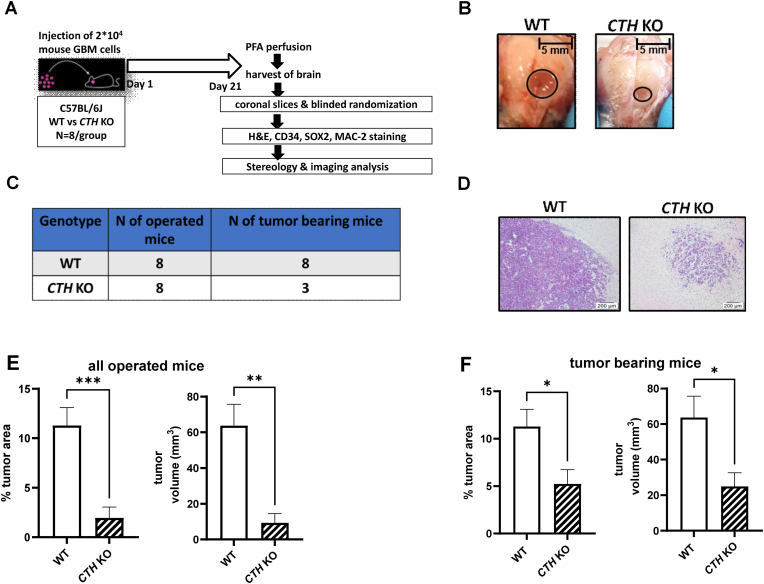
**Genetic ablation of *CTH* significantly attenuates glioblastoma formation in immunocompetent mice. A.** Overview of the *in vivo* experimental protocol. **B.** Representative macroscopic images of a WT and a *CTH* KO mouse 21 days after the stereotactic injection of the mouse GL261 GBM cells. **C.** Table comparing the number of operated vs tumor-bearing mice at the termination day in WT vs *CTH* KO mice. **D.** Representative microscopic images of a WT and *CTH* KO mouse GBM tumor after H&E staining, **E.** % of tumor area compared to the total brain area and the exact tumor volume in mm^3^ as calculated by stereology analysis for all operated mice (N = 8/group). Area as calculated by stereology analysis (N = 8/group). **F.** % of tumor area compared to the total brain area and the exact tumor volume in mm^3^ as calculated by stereology analysis for all the tumor-bearing mice (N = 8/group for WT and N = 3/group for *CTH* KO). Area as calculated by stereology analysis. *p<0.05, **p<0.01, ***p< 0.001, unpaired Student’s t-test.

Intracranial injection of mouse GBM GL261 cells expressing CTH (Supplemental Fig. 1) according to a well-established orthotopic immunocompetent mouse GBM model (Fig. 1A) led to substantially smaller tumors in *CTH* KO relative to WT mice. Details regarding the technical reliability of the surgeries and the number of the mice that developed visible by IHC tumors 3 weeks post-operation, are given in the Supplemental Table 2.

The differences in the tumor size were even seen macroscopically at the point of termination with the WT mice bearing obviously larger GBM tumors (Fig. 1B). As shown in Fig. 1C, all the operated WT mice developed visible tumors 21 days after the surgery whereas only 3 out of 8 *CTH* KO developed tumors under the same period. Indicative histology photographs from a WT and a *CTH* KO bearing tumors are shown in Fig. 1D.

The % of tumor area and tumor volume (mm^3^) was analyzed by blinded stereology in all the operated WT and *CTH* KO mice and showed substantially smaller tumors in the *CTH* KO mice both in terms of % tumor area and tumor volume (Fig. 1E).

The % of tumor area and the volume was also calculated by blinded stereology solely on the WT and *CTH* KO that developed tumors and we reached similar statistical conclusions indicating a significant reduction of the % tumor area and tumor volume in the *CTH* KO (Fig. 1F).

As a complementary measure, the % of tumor area and tumor volume were also measured by blinded microscopy, revealing similar/comparable to stereology findings (Supplemental Fig. 2 and Supplemental Table 3).

### 3.2 Genetic ablation of CTH in the mouse host does not lead to significantly altered levels of tumor microvascular density

**Fig. 2:**
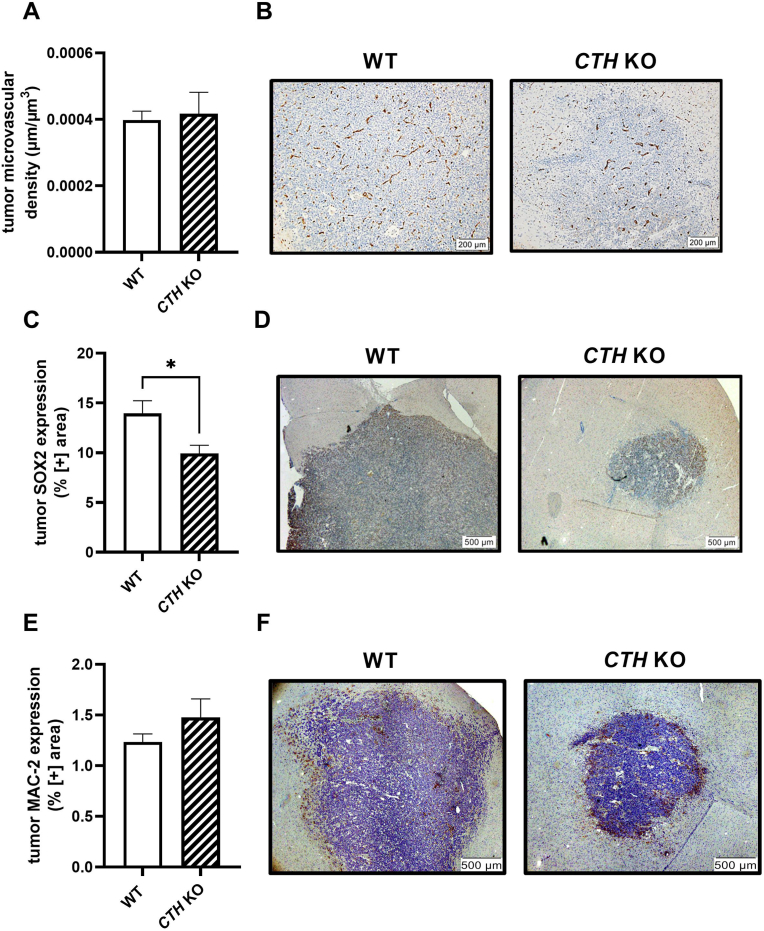
**Genetic ablation of *CTH* is associated with lower tumoral SOX2 (GSC marker) expression but does not affect the tumoral microvascular density (CD34) or macrophage levels (MAC-2). A.** Tumor microvessel density (μm of vessels/μm^3^ of tumor GBM tissue) in WT and *CTH* KO as calculated by stereology analysis (N = 8/group for WT, N = 3/group for *CTH* KO). **B.** Representative microscopic images depicting the tumor microvascular tree of a WT and a *CTH* KO mouse (hematoxylin/blue stains more intensely the tumor cells, CD34/endothelial cell marker in brown). **C.** Tumor SOX2 expression in WT and *CTH* KO mice as calculated by blinded microscopy analysis (N = 8/group for WT, N = 3/group for *CTH* KO). **D.** Representative microscopic images depicting the tumoral SOX2 expression of a WT and a *CTH* KO mouse (hematoxylin/blue stains more intensely the tumor cells, SOX2/GSC marker in brown). **E.** Tumoral MAC-2 expression in WT and *CTH* KO mice as calculated by blinded microscopy (N = 5/group for WT, N = 3/group for *CTH* KO). **F.** Representative microscopic images depicting the tumoral MAC-2 expression of a WT and a *CTH* KO mouse (hematoxylin/blue stains more intensely the tumor cells, MAC-2/general macrophage marker in brown). *p < 0.05, unpaired Student’s t-test. (For interpretation of the references to colour in this figure legend, the reader is referred to the Web version of this article.)

We used again, the blinded golden-standard method of stereological analysis and WT and *CTH* KO tumors were stained for CD34, a known endothelial cell marker which depicts nicely and reliably the microvascular network [29]. As shown in Fig. 2A and 2B the two genotypes had absolutely similar levels of tumor microvascular density indicating similar networks of blood vessels and degree of angiogenesis.

### 3.3 Genetic ablation of CTH in the mouse host leads to lower tumor SOX2 expression

Our IHC data and analysis of the same brain slices as the ones used above, but analyzed this time with blinded microscopy showed that *CTH* KO mice have a significantly lower tumoral SOX2 expression compared to WT bearing tumors (Fig. 2C and 2D).

### 3.4 Genetic ablation of CTH does not affect the number of tumor-associated macrophages (TAMs)

MAC-2 (Galectin-3) was used as a general pan-macrophage marker [30] and IHC analysis was performed by blinded microscopy, using WT and *CTH* KO-derived tumors. Our results showed no significant differences on tumoral MAC-2 expression between the two genotypes (Fig. 2E and 2F).

### 3.5 CTH is overexpressed in human GBM and correlates positively with SOX2 expression but not with EC markers

**Fig. 3:**
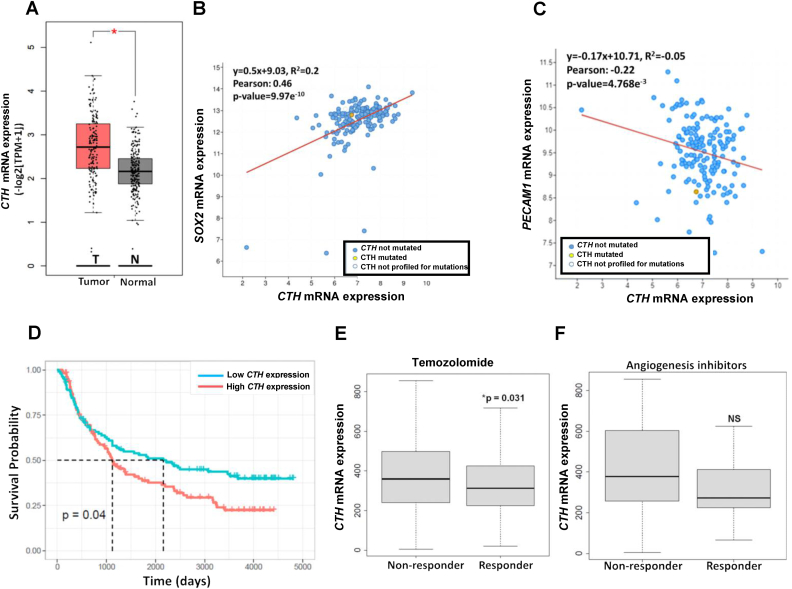
**Higher *CTH* expression in human gliomas is associated with worse overall survival, higher *SOX2* expression and non-response to temozolomide but is not positively correlated with the expression of endothelial cell markers or response to anti-angiogenic treatments. A.***CTH* mRNA (TCGA database) expression is significantly enhanced in human GBM tumors (T) compared to healthy/non-GBM (N) brain tissue. **B.***CTH* mRNA expression is positively correlated with *SOX2* mRNA expression. **C.***CTH* mRNA expression in GBM tumors is not positively associated with the mRNA expression of the endothelial cell marker *PECAM1*. **D.** GBM patients with higher tumor *CTH* mRNA expression levels have significantly lower survival probability in all grade primary gliomas compared to GBM patients with lower *CTHmRNA* expression (CGGA atlas). **E, F:** Higher *CTH* mRNA expression is significantly associated with non-response to temozolomide (**E**) but not with anti-angiogenesis inhibitors **(F)**: bevacizumab, thalidomide, vandetanib, vatalanib. The Cancer Genome Atlas (TCGA) and Genotype-Tissue Expression (GTEx) datasets were used to assess the expression of *CTH* in GBM compared to normal brain tissue using GEPIA2 (http://gepia2.cancer-pku.cn) (panel **A**). The cBioPortal for Cancer Genomics was used to retrieve RNA-seq data from GBM patients and gene coexpression analyses of *CTH* with other genes of interest. batch normalized from Illumina HiSeq_RNASeqV2, blue dots: non-mutated *CTH*, yellow dots: mutated *CTH*, white dots: *CTH* not profiled for mutation (panels **B, C**). The Chinese Glioma Genoma Atlas (CGGA) was used to retrieve mRNA microarray and RNA-seq data of GBM patients and generate survival Kaplan-Meier plot (panel **D**). Correlation of gene expression with treatment response to temozolomide and angiogenesis inhibitors in GBM patients was conducted using the database from ROC Plotter (https://www.rocplot.org/gbm). GBM patients were categorized as responders or non-responders, according to the survival status at 16 months’ post-surgery, and gene expression was compared with the Mann-Whitney *U* test. (For interpretation of the references to colour in this figure legend, the reader is referred to the Web version of this article.)

Based on bioinformatic analysis on ‘The Cancer Genome Atlas’, TCGA, we found a significant upregulation of *CTH* mRNA expression in all grades of human gliomas compared to healthy/non-tumoral brain tissue (Fig. 3A). Our database analysis on RNAseq from TCGA database also revealed a significant positive correlation between *CTH* and the GSC marker *SOX2* (mRNA expression) in human GBM (Fig. 3B). However, there was no positive correlation between *CTH* and the mRNA levels of the well-established EC marker *PECAM1/CD31* (Fig. 3C). Finally, there was no correlation between *CTH* and the mRNA levels of other well-established GSC markers such as *NES, PROM1* or *CD44* (Supplemental Figs. 3A–C) or the EC marker *CLDN5* (Supplemental Fig. 3D).

### 3.6 CTH overexpression is associated with worse overall survival in primary gliomas

By analyzing the *CTH* mRNA expression profile on the CGGA database, we found that higher *CTH* expression is correlated with significantly worse survival probability in all primary gliomas of different grades (Fig. 3D). Moreover, high *CTH* expression led to a clear trend with almost acceptable significance (P-value: 0.056) for lower survival probability in recurrent gliomas of all grades (Supplemental Fig. 3E).

### 3.7 Non-responders to temozolomide (TMZ) have higher *CTH* expression

In order to further evaluate the clinical relevance and the pharmacological potential of our findings, we used the www.rocplot.org database, which is capable to link gene expression and response to therapy using transcriptome-level data of human GBM. Our analyses revealed that higher *CTH* mRNA expression in human GBM correlates significantly with non-response to temozolomide (Fig. 3E), which is the most commonly used current chemotherapy against GBM [31].

### 3.8 CTH expression does not differ between responders and non-responders to angiogenesis inhibitors

Our analysis revealed that the higher *CTH* expression did not correlate significantly to non-responder patients who received approved anti-angiogenic treatments such as bevacizumab, thalidomide, vandetanib and vatalanib (Fig. 3F).

### 3.9 Pharmacological inhibition of CTH leads to lower stem cell frequency and expression of stem cell markers on human GBM cells

**Fig. 4:**
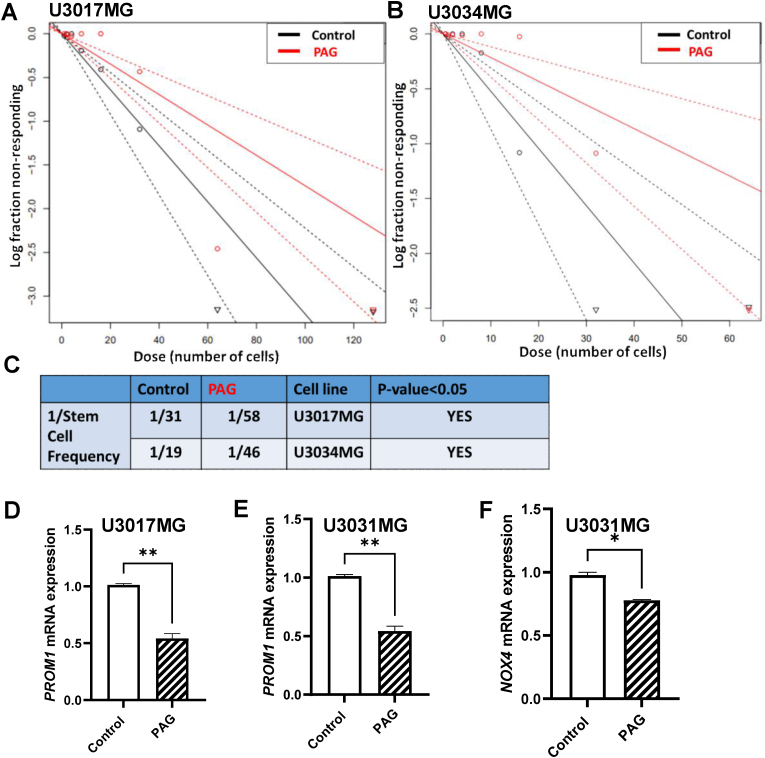
**A brain permeable pharmacological inhibitor of CTH attenuates the formation of human glioblastoma stem cells (GSC) and the expression of GSC markers. A, B.** The brain permeable CTH pharmacological inhibitor PAG (600 μΜ, 8 days) attenuated significantly the stem cell frequency formation of human GSC in two different human glioblastoma lines (U3017MG and U3034MG) as accessed by ELDA, N = 6/group, **C**. 1/Estimated cell frequency in numerical values for the Control- and PAG-treated cells of panels A and B. **D, E:** mRNA expression levels of the GSC marker *PROM1* in two different human glioblastoma lines (U3017MG, U3031MG). N = 2/group (average of 2 biological replicates) **F:** mRNA expression levels of the GSC marker *NOX4* in human glioblastoma cells (U3031MG), N = 2/group (average of 2 biological replicates). *p < 0.05, **p<0.01, unpaired Student’s t-test.

As shown in Fig. 4A–C, the treatment with a non-cytotoxic PAG concentration (600 μΜ) was able to significantly reduce the stem cell frequency on two different human GBM cell lines. At the molecular level, we tested the mRNA expression of the stem cell markers *PROM1* and *NOX4* and we found a significant downregulation upon CTH inhibition (Fig. 4D–F). Interestingly, direct pharmacological inhibition of CTH on mouse and human GBM cells does not alter the expression levels of the GSC marker SOX2 (Supplemental Fig. 4).

### 3.10 Pharmacological (PAG) CTH inhibition or CTH knockdown (siRNA) leads to lower mouse GBM cell proliferation and migration via an H_2_S-dependent mechanism

**Fig. 5:**
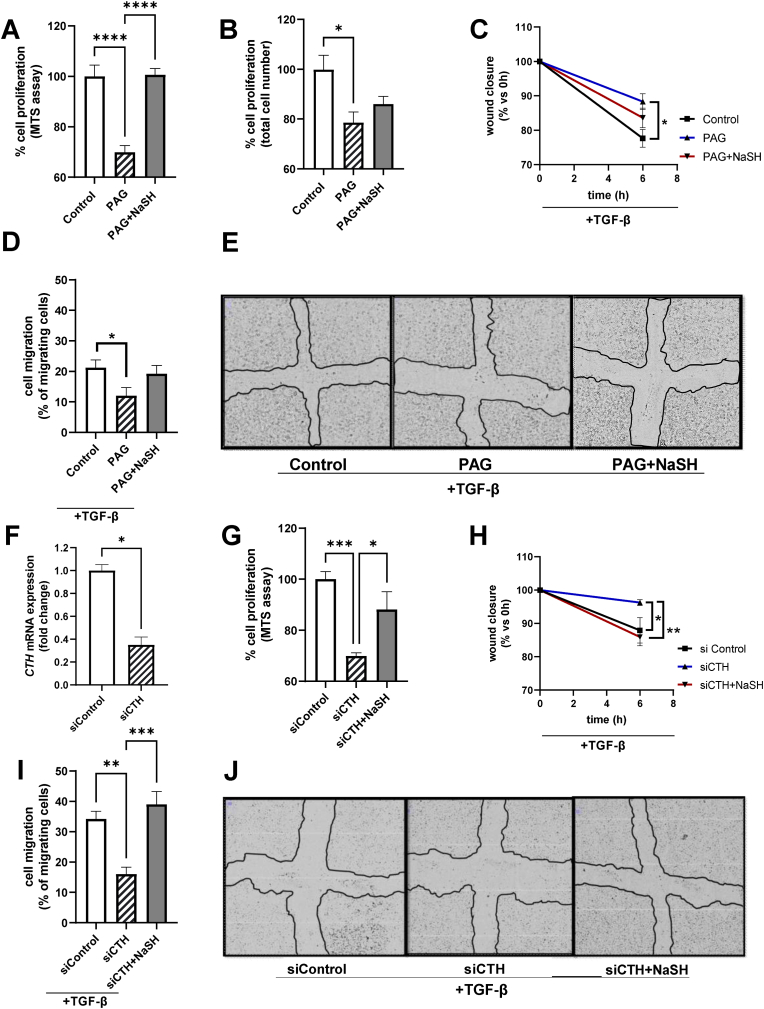
**Pharmacological inhibition (PAG) or CTH knockdown (siRNA) attenuates mouse GBM cell proliferation and migration via an H**_**2**_**S-dependent mechanism. A, B:** % of cell proliferation of Control (0.1% DMSO, 24 h), PAG (600 μΜ, 24 h) and PAG (600 μΜ, 24 h)+NaSH (150 μΜ, 24 h) treated cells as assessed by the MTS assay (**A**) and by Trypan Blue staining and counting of the total alive cell number (**B**). N = 6/group for (**A**) and N = 4/group for (**B**). **C:** Wound closure over time (% of closure area of 0 vs 6 h) for Control (0.1% DMSO, 6 h), PAG (600 μΜ, 6 h) and PAG (600 μΜ, 6 h)+NaSH (150 μΜ, 6 h) treated cells. N = 3/group **D:** % of migrating cells (migrating cells into the ‘cross/scratch’ area from 0 vs 6 h) for Control (0.1% DMSO, 6 h), PAG (600 μΜ, 6 h) and PAG (600 μΜ, 6 h)+NaSH (150 μΜ, 6 h) treated cells. N = 6/group **E:** Representative microscopic images at the 6h time point for Control (0.1% DMSO), PAG (600 μΜ) and PAG (600 μΜ)+NaSH (150 μΜ) treated cells. The black drawn line in the middle depicts the remaining cell-free area 6 h after scratching. **F:***CTH* mRNA expression for siControl (treated with scrambled ‘Control’ siRNA, 30 nM, 24 h) and siCTH (treated with siRNA for CTH, 30 nM, 24 h), N = 2 biological replicates/group. **G:** % of cell proliferation of siControl (scrambled ‘Control’ siRNA, 30 nM, 24 h), siCTH (siRNA for CTH, 30 nM), and siCTH (siRNA for CTH, 30 nM)+NaSH (150 μΜ, 24 h) treated cells as assessed by the MTS assay, N = 12/group **H:** Wound closure over time (% of closure area of 0 vs 6 h after scratching) for siControl (scrambled ‘Control’ siRNA, 30 nM), siCTH (siRNA for CTH, 30 nM) and siCTH (siRNA for CTH, 30 nM)+NaSH (150 μΜ) treated cells. N = 3/group **I:** % of migrating cells (migrating cells into the ‘cross/scratch’ area from 0 vs 6 h after scratching) for siControl (scrambled ‘Control’ siRNA, 30 nM), siCTH (siRNA for CTH, 30 nM) and siCTH (siRNA for CTH, 30 nM)+NaSH (150 μΜ) treated cells. N = 6/group. **J:** Representative microscopic images at the 6 h time point for siControl (scrambled ‘Control’ siRNA, 30 nM), siCTH (siRNA for CTH, 30 nM) and siCTH (siRNA for CTH, 30 nM)+NaSH (150 μΜ) treated cells. The black drawn line in the middle depicts the remaining cell-free area 6 h after scratching. Comparisons between 3 groups in panels **A, B, D, G** and **I** were performed by one-way ANOVA followed by Bonferroni’s multiple comparisons test. Comparisons between 3 groups over time (panels **C, H**) were performed by two-way ANOVA followed by Bonferroni’s multiple comparisons test. Comparisons between 2 groups (panel **F**) was performed by unpaired Student’s t-test. **Special notes:** PAG was added 30 min prior any other treatment. Cells were transfected with siRNA at the moment of plating (reverse transfection) and therefore were treated with the respective siRNA for at least 24h before the addition of any other reagent (i.e NaSH, TGF-β). For the cell migration assays (panels **C, D, E, H, I, J**) all the cells received an equal amount of the cell migration-promoting factor TGF-β1 (5 ng/ml). (For interpretation of the references to colour in this figure legend, the reader is referred to the Web version of this article.)

Pharmacological inhibition of CTH with PAG led to significantly lower cell proliferation, an effect partially or fully reversed by the H_2_S donor, NaSH (Fig. 5A–B). At the same time, various concentrations of PAG did not have any major cytotoxic effect as tested in different mouse and human GBM cell lines (Supplemental Fig. 5). Administration of the H_2_S donor, NaSH alone (without PAG) did not have any major cytotoxic effect either (Supplemental Fig. 6A), whereas it had at the same time a mild attenuating effect on cell proliferation (Supplemental Fig. 6B).

TGF-β is known as a strong stimulator of GBM cell migration and invasiveness [32]. As shown in the Supplemental Fig. 7A, our findings confirmed previous results and showed that TGF-β promoted GBM cell migration, an effect reversed by PAG. Moreover, PAG attenuated the TGF-β induced mediated H_2_O_2_ production/NOX4 activity (Supplemental Fig. 7B), which is a known stimulator of cell migration and invasiveness [33].

In order to further expand on these findings and investigate if the effects of CTH are H_2_S-dependent, we measured the levels of wound closure and cell migration by performing scratch assays in the presence of PAG ± NaSH. As shown in Fig. 5C–E, PAG significantly hindered the TGF-β induced cell migration, an effect partially reversed by the H_2_S donor, NaSH.

Lastly, siRNA against the mouse *CTH* mRNA was used and cell proliferation and migration were measured. Administration of 30 nM siRNA against CTH (indicated as siCTH) downregulated significantly the *CTH* mRNA expression at about ∼70% (Fig. 5F) and this concentration was used for subsequent cell proliferation and migration assays. As shown in Fig. 5G, siCTH significantly reduced the cell proliferation levels, an effect reversed by the H_2_S donor, NaSH. Moreover, siCTH attenuates significantly the TGF-β induced cell migration, an effect fully reversed by the H_2_S donor, NaSH (Fig. 5H–J).

## 4 Discussion

Elevated levels of H_2_S by biosynthetic routes such as the enzymes CTH, CBS or 3-MST seem to play an important role in supporting tumorigenesis [34]. In relation to glioblastoma formation, CBS overexpression seems to attenuate rather than supporting gliomagenesis. A study published in 2014 [35] showed that decreased expression of CBS promotes gliomagenesis. Therefore, it is rather the activation and not the inhibition of CBS that could potentially play an anti-tumoral effect in glioblastoma. Moreover, Silver et al., 2021 showed that High Fat Diet predisposes mice for formulating more aggressive GBM tumors partially because of lower CBS expression [36]. On the contrary, a study published last year showed that 3-MST expressed in the glioblastoma cells supports gliomagenesis and therefore 3-MST inhibition could have a potential promising therapeutic effect against glioblastoma [37].

A study published last year showed that CTH is upregulated in IDH-1 mutant astrocytomas and pharmacological CTH inhibition could attenuate to some extent astrocytoma growth in immunodeficient mice [12]. According to the latest (2021) WHO classification, IDH-1 mutant astrocytomas have a more favorable prognosis and cannot be longer classified as GBM [38]. The role of CTH in IDH-1 WT GBM has not been extensively investigated. In particular, the exact biological role of CTH expressed in the tumor stroma (microenvironment) vs the GBM tumor cells per se remains to a large extend unknown especially under the setting of immunocompetent mice*.* To the best of our knowledge, our study is the first one investigating in detail these aspects.

Targeting the GBM cells per se pharmacologically, remains up to date, a very difficult task since the GBM cells have a very aggressive phenotype [39]. Even strong cytotoxic reagents such as temozolomide (TMZ) combined with radiotherapy that target the GBM cells and prolong survival [40] are eventually to a large extent insufficient since in the majority of GBM patients there is a recurrence of tumor formation and most of the patients die within 5 years from the time of the first diagnosis [5]. Therefore, identifying new pharmacological targets in the tumor microenvironment and not only in the tumor cells per se could be a new promising therapeutic approach for the battle against GBM.

In this study, we hypothesized that inhibiting CTH in the tumor microenvironment could have potentially anti-tumoral effects since similar effects of CTH have been shown in other cancer types [34]. Moreover, we hypothesized that direct CTH inhibition on mouse and human GBM cells could have extra anti-tumoral functions, aiding and supporting the anti-tumoral effect of CTH inhibition in the tumor stroma.

Since CTH is a known inducer of angiogenesis [8] and the vascular network is known to play a crucial role in supporting glioblastoma formation [3], we wanted first to determine if the substantially smaller tumors observed in the *CTH* KO were also accompanied by an altered tumor microvascular network.

Analyzing the tumor microvascular density by stereological analysis is a well-established method that reveals with high accuracy and reliability the levels of tumor vasculature and when differences in the tumor microvascular density are observed, this is indicative of different levels of angiogenesis [41]. Our results indicate similar levels of tumor microvascular density between the WT and the *CTH* KO mice.

Therefore, despite the fact that in other cancer models [10,11], CTH has been shown to promote tumor angiogenesis, this does not seem to be the case for glioblastoma formation, at least not due to CTH expression in the tumor microenvironment, in this well-established orthotopic immunocompetent mouse GBM [15]. Future studies with the use of human GBM cells on xenografted mice will be necessary to further support our current findings and test rigorously whether CTH acts independently of angiogenesis when GBM tumors are formed by human cells. However, recent GBM xenograft studies should be interpreted cautiously since the host mice were immunodeficient [42], and the immune system is known to play a crucial role in supporting tumor angiogenesis [43].

Moreover, our bioinformatic analysis on human GBM gene expression data, further supports our *in vivo* findings showing that *CTH* expression in human GBM tumors is not associated with the expression of EC markers or with the patients’ response to anti-angiogenic treatments.

Whether CTH inhibition in the tumor cells per se but not in the stroma, could have an effect on angiogenesis, remains to be elucidated. Future pre-clinical and clinical trials will show whether pharmacological inhibitors of CTH can enhance the anti-tumoral activity of temozolomide or other chemotherapies against GBM.

Apart from the tumoral endothelium, the tumor-associated macrophages (TAMs) comprise another major component of the tumor microenvironment that supports glioblastoma formation [44]. TAMs are the dominant infiltrating immune cell population in GBM and they aid glioblastoma formation by different mechanisms, like supporting GSC formation [45] or inducing neovascularization [43]. Our results showed no significant differences on the levels of TAMs between the WT and *CTH* KO mice. Thus, we could hypothesize that the CTH expression in the tumor microenvironment is not primarily responsible for driving macrophage infiltration inside the glioblastoma tumors.

However, future studies are needed in order to elucidate if there are any differences in tumor macrophage subpopulations (i.e M1 and M2) or in the numbers of other immune cell types that infiltrate the tumor microenvironment, and whether CTH might be involved in such changes.

Since lower levels of tumoral angiogenesis and tumoral macrophage infiltration does not seem to be the main players driving the attenuated glioblastoma formation in the CTH KO mice, we next decided to focus on molecular players driving glioblastoma stem cell (GSC) formation. GSC formation and self-renewal is one of the major drivers of gliomagenesis that it is controlled by several autocrine and paracrine signals derived either from the tumor cells per se or from the tumor stroma [46].

SOX2 is a master transcription factor of mammalian cell pluripotency and stemness that also regulates the expression of several genes promoting self-renewal of glioblastoma stem cells (GSCs) [47]. Moreover, SOX2 has been shown to promote malignancy in GBM by regulating the plasticity and differentiation fate not only of GCSs but also of differentiated astrocytes [48].

Interestingly, bio-informatic analysis performed by us in the current study revealed a positive and significant correlation between *CTH* and *SOX2* mRNA expression in human GBM tumors but whether this correlation is driven by the tumor microenvironment, or the tumor cells alone remained unknown. To answer this question, the tumoral SOX2 expression levels were evaluated *in vivo* in the *CTH* KO and in isolated mouse and human GBM cells where CTH was pharmacologically inhibited.

Our combined *in vivo* and cellular findings indicate that SOX2 tumoral protein expression is significantly downregulated in the *CTH* KO but remains unchanged upon direct CTH inhibition on mouse and human GBM cells.

Our findings therefore suggest that the absence of CTH in the tumor microenvironment and not in the tumor per se, is sufficient for regulating tumoral SOX2 expression. The exact cellular sources and nature of the paracrine signals leading to this outcome remain to be identified in future studies. The cellular organization of the GBM microenvironment is extremely complex with many different cell types i.e endothelial cells, macrophages, astrocytes, microglia etc, being involved [3]. In order to identify the most important cell type(s) leading to lower tumoral SOX2 levels in the *CTH* KO, a series of tumor microenvironment cell-specific *CTH* KO mice would have to be created (i.e knockout in endothelial cells, macrophages, astrocytes) which was beyond the scope of the current study.

In general, GBM patients with higher GSC gene expression profile, including SOX2, have worse prognosis and overall survival [49]. Since SOX2 overexpression is linked to worse overall survival in human GBM [49] and the expression of this protein is positively correlated to *CTH* expression, we next sought to investigate if *CTH* overexpression is linked to worse survival in humans GBM patients. Indeed, our bioinformatic analysis suggests that *CTH* is overexpressed in human GBM tumors and higher *CTH* expression is significantly correlated with lower survival probability in all grades of primary gliomas. Moreover, there was an almost significant trend in all grades of recurrent gliomas. In conclusion, our results might indicate that *CTH* overexpression by supporting GBM formation, leads to lower survival probability in humans.

We must however point out that correlation findings should be interpreted with caution and not as a proof-of-concept. Future detailed clinically-oriented studies are needed in order to understand the role and the expression profile of CTH in human GBM pathogenesis. An additional reason why the bioinformatic data should be interpreted with caution from the point of view of this study, is the lack of data that distinguish gene expression in the tumor cells versus the tumor stroma. Future single cell analysis in GBM will be valuable in resolving this problem and will offer better data for the evaluation of our GBM model in the *CTH* KO mouse.

Finally, and in order to clarify if direct CTH inhibition on the tumor cells could trigger any additional anti-tumoral effects, human and mouse GBM cells were treated with pharmacological (PAG) or molecular (siRNA) CTH inhibitors. Biological phenomena critical for glioblastoma formation, maintenance and recurrence such as cell viability, cell proliferation, cell migration and stem cell formation [[50], [51], [52]] were measured.

Our results show that the brain-permeable pharmacological inhibitor of CTH, PAG, is not a cytotoxic drug but can attenuate significantly the GBM cell proliferation and the TGF-β-induced cell migration in mouse glioblastoma cells by an H_2_S-dependent mechanism. Moreover, PAG can attenuate the TGF-β-induced NOX4-derived ROS which are in general known to lead to genomic instability and trigger signaling cascades promoting cell migration and invasion in cancer [53]. Indeed, a recent publication from our group indicated that NOX4 activity and expression is significantly triggered by TGF-β1 signaling in different GBM lines promoting stemness and proliferation [14].

Additionally, PAG under baseline conditions can significantly reduce the formation of glioblastoma stem cells derived from human GBM cells and downregulate the mRNA expression of genes involved in stem cell formation such as PROM1 and NOX4 [14,54,55].

## 5 Conclusions-importance of the study

In conclusion, the present study **emphasizes the importance of CTH as a regulator of glioblastoma formation both in the tumor microenvironment and in the GBM cells per se.**

So far **there has not been any genetic studies using *CTH* KO immunocompetent mice,** which would enable us to understand whether targeting CTH in the tumor microenvironment is sufficient to suppress glioblastoma formation. Our results showed **markedly attenuated glioblastoma formation in immunocompetent *CTH* KO mice** bearing CTH-expressing tumors. Therefore, **CTH expressed in the tumor stroma is an important positive regulator of glioblastoma** formation and targeted administration of CTH pharmacological inhibitors in the tumor stroma might reveal in the future a promising therapeutic potential.

Our findings also suggest a potential **link between CTH and molecular players regulating GBM stemness** acting both via cells in the tumor microenvironment, and in the tumor cells per se. Finally, CTH expressed in the mouse GBM cells is a **positive regulator of cell proliferation and cell migration by H**_**2**_**S-dependent mechanisms.** Future studies are needed in order to point out the exact cellular and molecular players in this process and the potential of CTH pharmacological or RNA-based inhibitors in the battle against glioblastoma. Our bioinformatic analysis **shows that higher *CTH* expression occurs in patients with gliomas and is associated with lower survival probability.**

**Therefore, our findings open a new chapter not only towards glioblastoma treatment, but equally important, provide novel molecular tools for the prognostic analysis of this disease**.

A summary of the proposed mechanism(s) is shown **in the Graphical abstract**.

## Funding

This work was funded by the 10.13039/501100004359Swedish Research Council International Postdoc Grant (Vetenskapsrådet, Grant number: 2019-00534) to M.P and the 10.13039/501100002794Swedish Cancer Society (CAN2018/469, CAN2021/1506Pj01H) to A.M. D.F.J.K is supported by grants from the 10.13039/501100009708Novo Nordisk Foundation (MeRIAD consortium, 0064142), and the 10.13039/501100006356University of Southern Denmark.

## Authorship statement

All authors have made substantial contributions to the manuscript as indicated in detail below. Moreover, all authors were involved in drafting the article or revising it critically for important intellectual content and in the final approval of the version to be submitted.

**Conceptualization**: M.P, A.M, A.P **Methodology:** M.P, I.A, D.M.R.J, O.S, A.K **Investigation**: M.P, I.A, D.M.R.J, O.S, K.M, **Formal analysis:** M.P, I.A, D.M.R.J, O.S, K.M, **Writing – Original Draft:** M.P, I.A, D.R.M.J, L.C, A.K, D.F.J.K, J.S, K.M, A.M, A.P, **Writing – Review and Editing**: M.P, D.M.R.J, L.C, A.K, A.M, A.P, **Funding acquisition:** M.P, A.M, D.F.J.K **Resources:** M.P, A.M, A.P, D.F.J.K, **Supervision:** A.M, A.P.

## Declaration of competing interest

The authors declare no conflict of interest.
