# Supplementary figures and images for: Cystathionine gamma-lyase (CTH) inhibition attenuates glioblastoma formation
Source: Redox Biol. 2023 Jun 5;64:102773. doi: 10.1016/j.redox.2023.102773 (PMC10363444; doi:10.1016/j.redox.2023.102773)

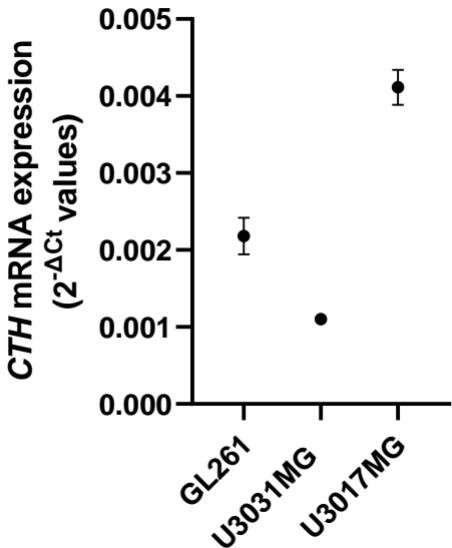

Supplement: Multimedia component 1 [file mmc1.pdf]

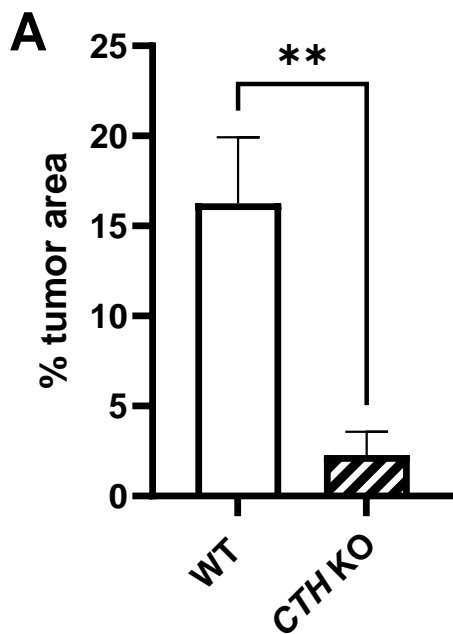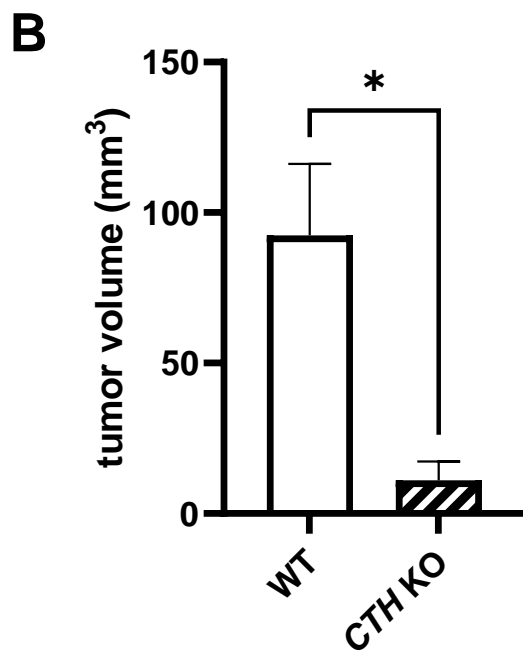

*Blinded Microscopy all  
operated mice*

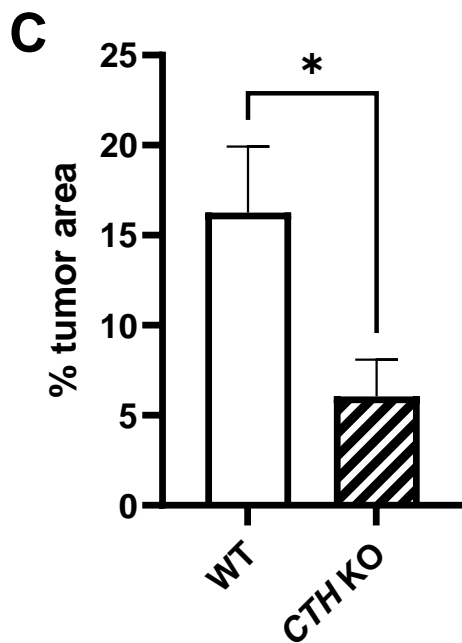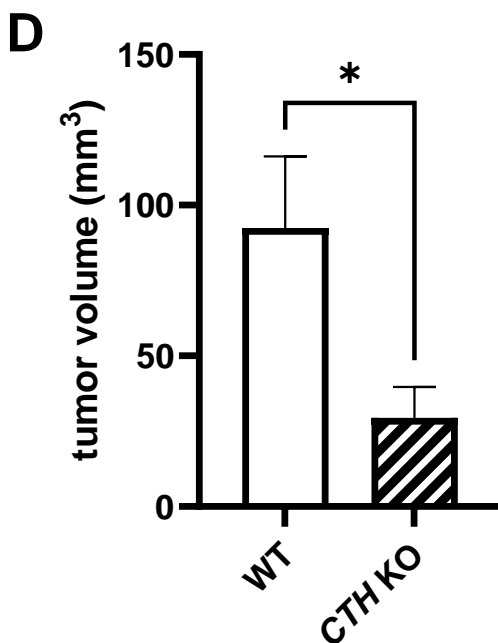

*Blinded Microscopy  
tumor bearing mice  
on termination*

Supplement: Multimedia component 2 [file mmc2.pdf]

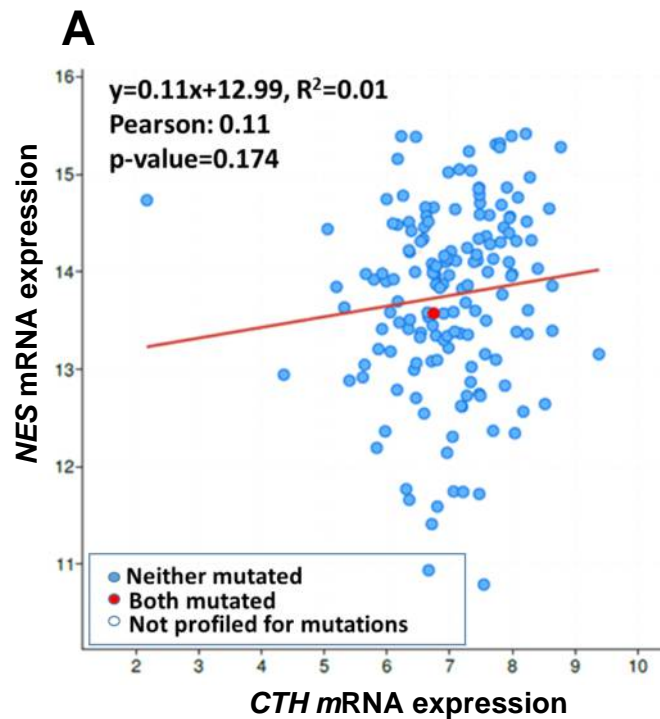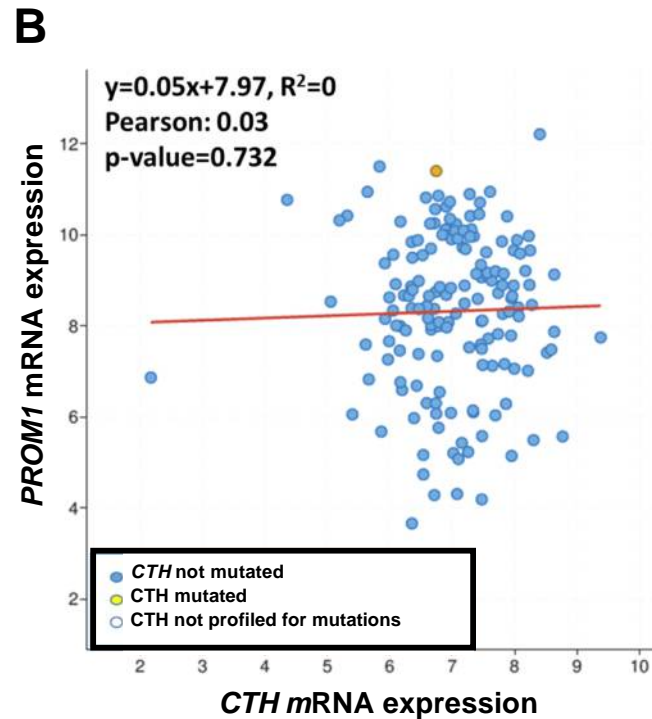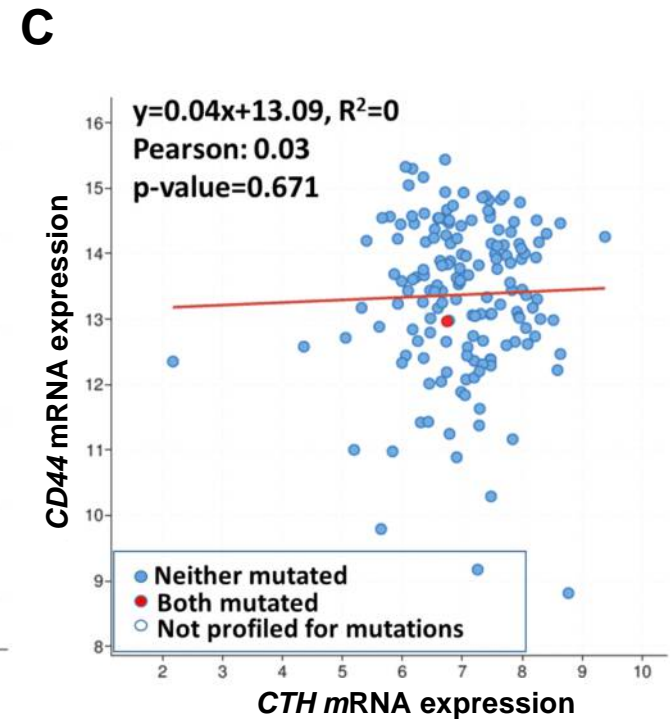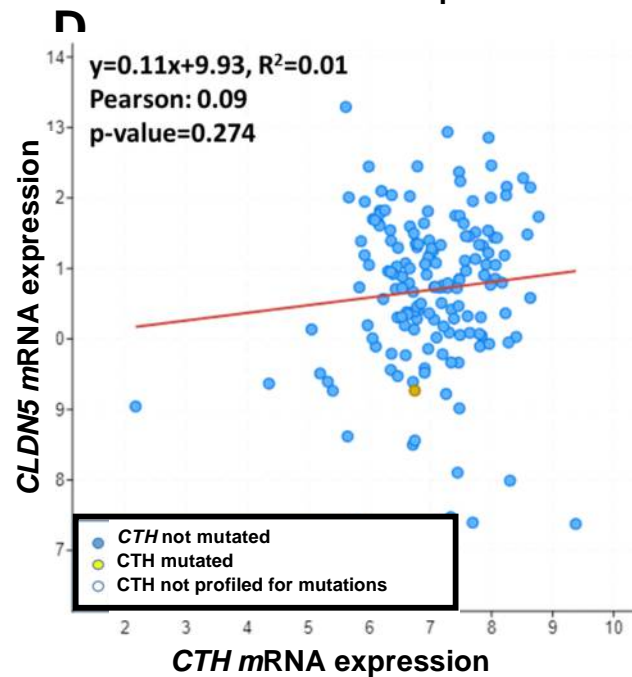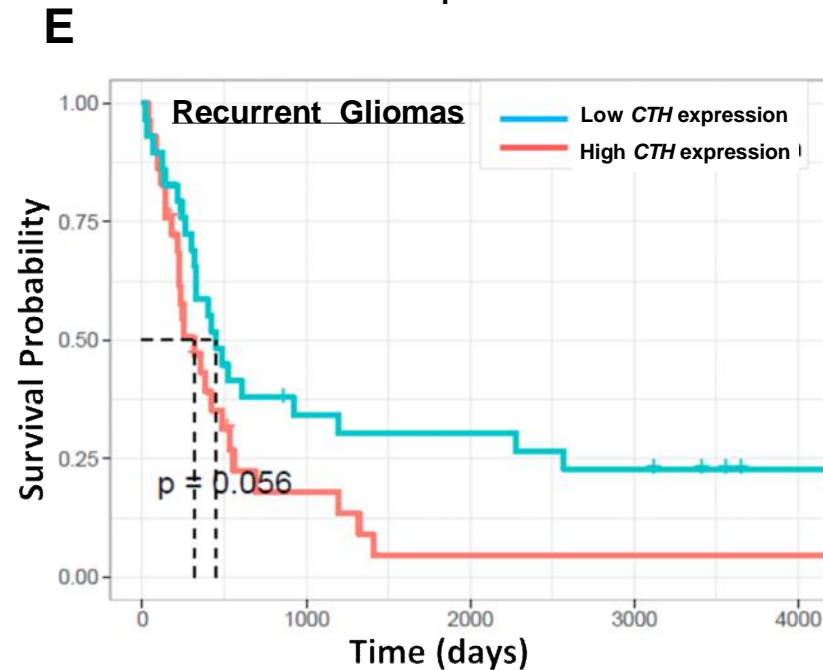

Supplement: Multimedia component 3 [file mmc3.pdf]

**A**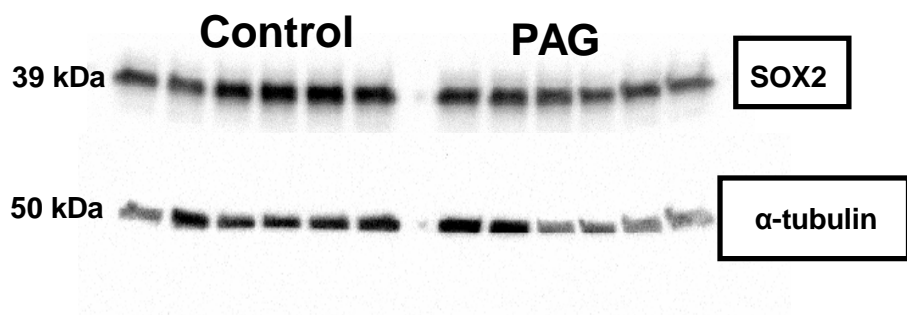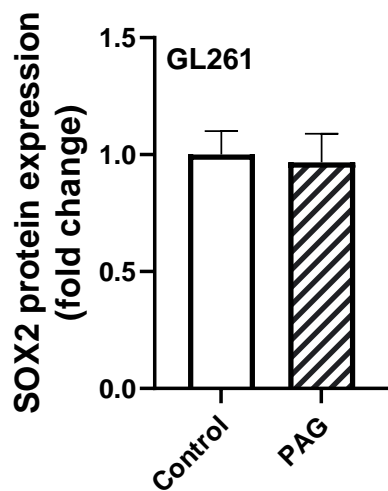**B**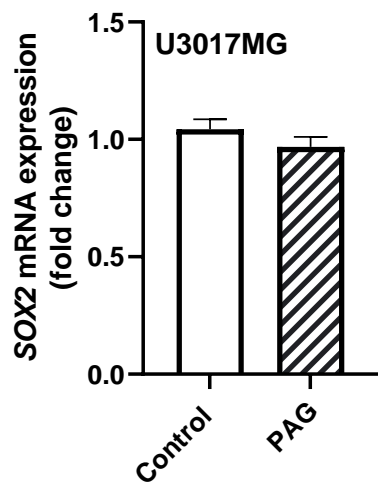**C**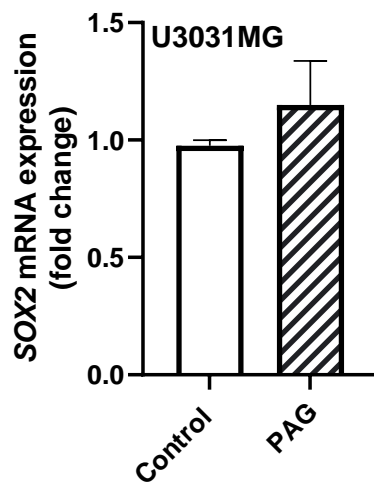

Supplement: Multimedia component 4 [file mmc4.pdf]

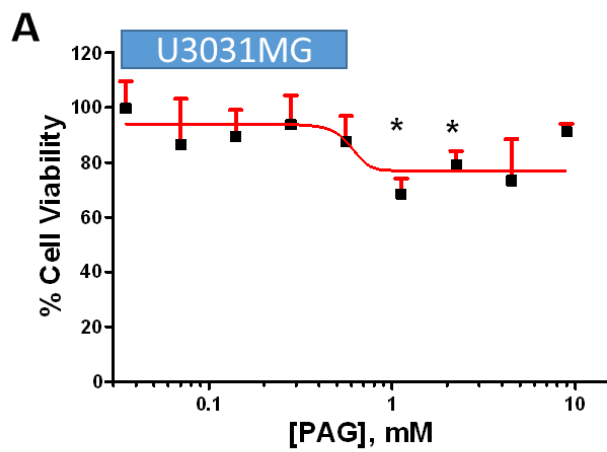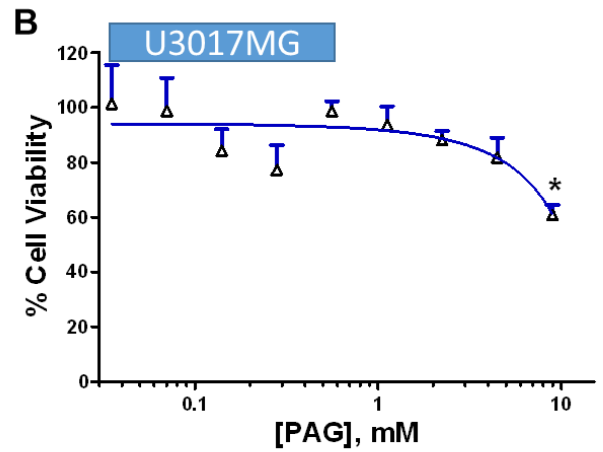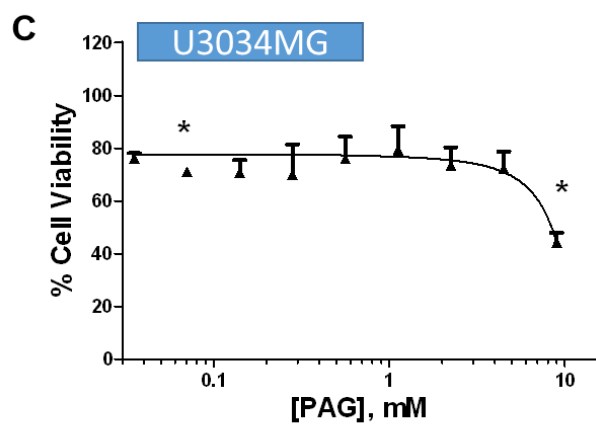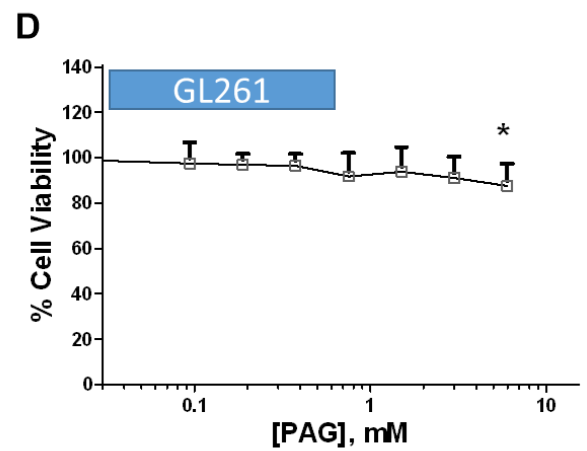

Supplement: Multimedia component 5 [file mmc5.pdf]

**A**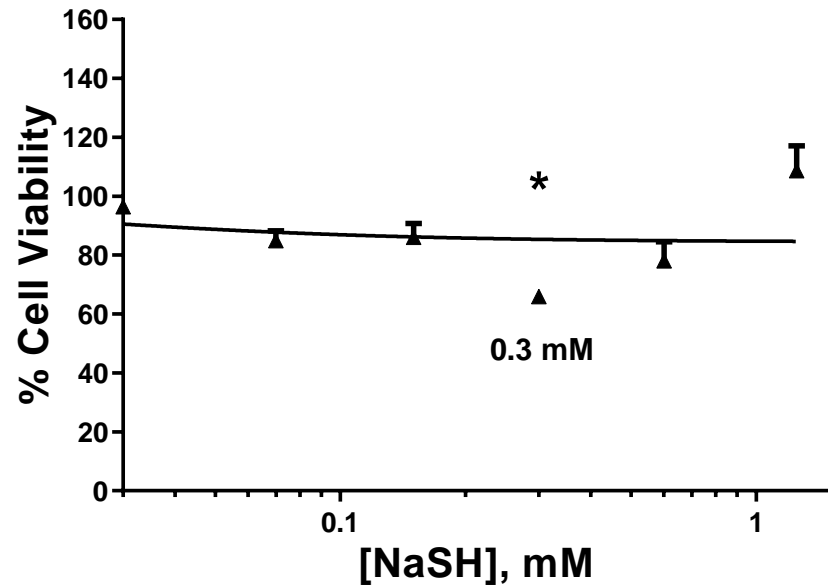**B**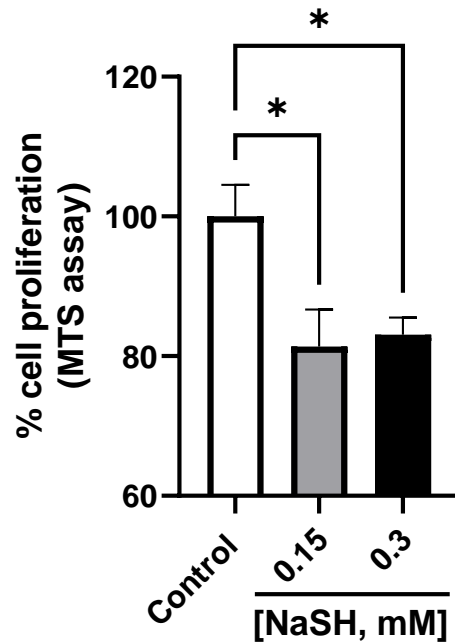

Supplement: Multimedia component 6 [file mmc6.pdf]

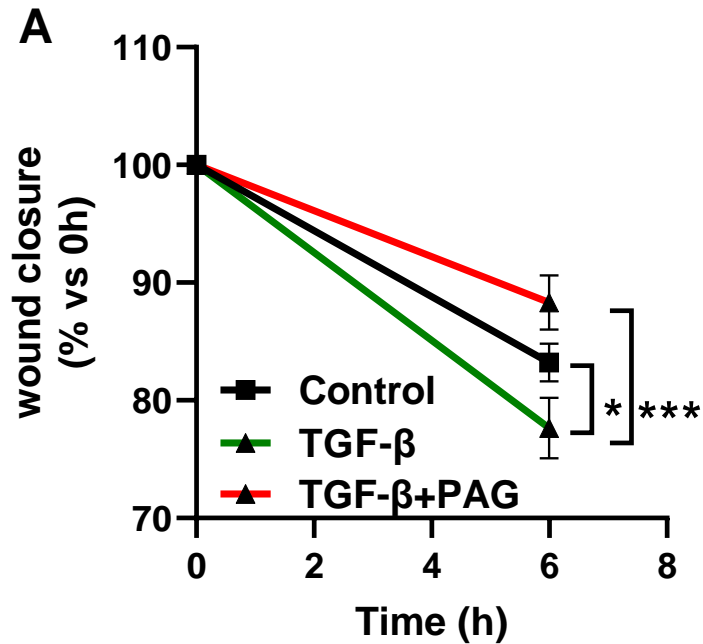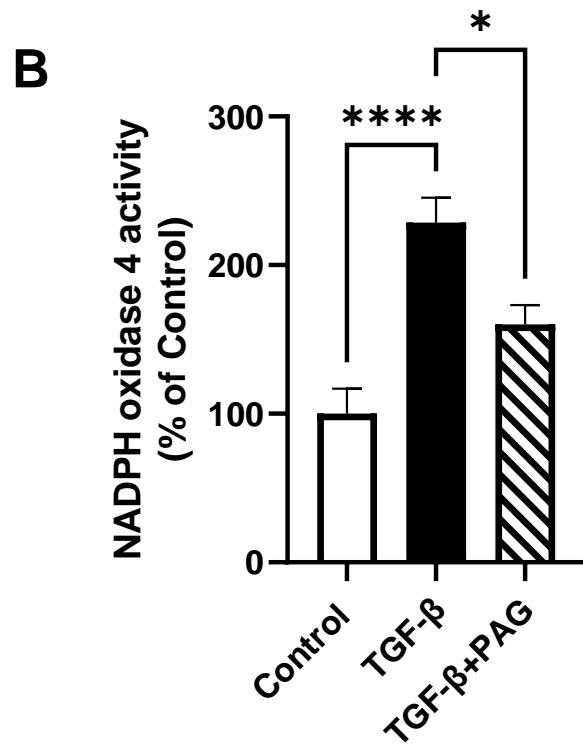

Supplement: Multimedia component 7 [file mmc7.pdf]
