# Supplementary material for: Cystathionine gamma-lyase (CTH) inhibition attenuates glioblastoma formation
Source: Redox Biol. 2023 Jun 5;64:102773. doi: 10.1016/j.redox.2023.102773 (PMC10363444; doi:10.1016/j.redox.2023.102773)
Supplement: Multimedia component 8 [file mmc8.docx]

**Supplemental Information on ‘Materials and Methods’**

**Cell Culture**

The human GBM lines were cultured at 37 °C (5% CO_2_ and 100% humidity) under serum-free conditions using the N2B27 medium. The N2B27 medium was composed of DMEM/F12 (50% v/v), Neurobasal (50% v/v), B27 without vitamin A (2% v/v), N2 (1% v/v) (Thermo Fisher Scientific, Waltham, MA, USA), Glutamine (1% v/v), Penicillin–Streptomycin (1% v/v) (Sigma-Aldrich Co., St. Louis, MO, USA); the N2B27 medium was supplemented with EGF and bFGF (10 ng/mL both, Peprotech, Rocky Hill, NJ, USA). The GL261 murine glioma cell line was a gift from and authenticated by Prof.Anna Dimberg, Dept.of Immunology, Genetics and Pathology, Uppsala University, Uppsala, Sweden, using PCR-single locus technology (Eurofins, Uppsala, Sweden). The GL261 cells were cultured in Dulbecco’s modified Eagle’s medium (61695, Life Technologies Gibco) with 10% heat-inactivated fetal bovine serum (F7524, Sigma). All GBM cell cultures were free of mycoplasma (tested every 3 months).

**MTS (Cell Viability) Assay**

GL261, U3031MG, U3037MG and U3034MG cells were seeded in a 96-well plate and the cell viability was assessed by using the MTS assay. The next day after seeding, the cells were treated with different concentrations of PAG for 48h. A wide range of PAG concentrations were tested and we observed that PAG did not exhibit strong cytotoxicity. At the (1-10) mM range PAG could reduce significantly the % of cell viability in some of the tested GBM lines, however even at this high PAG concentration range, the levels of cell viability were 45-50% or higher, indicating that PAG is not primarily a cytotoxic drug. Our results expand further and agree with a recent publication which also showed that PAG under normal/physiological cysteine concentrations does not have a strong cytotoxic effect in astrocytoma cells [1].

**Orthotopic GL261 mouse GBM models**

8 week-old female C57BL/6 WT and *CTH* KO mice housed in the animal facility of the Biomedical Research Foundation Academy of Athens (BRFAA, Athens, Greece) were used in this study. The genotype of the mice was confirmed for all individual mice by genotyping as previously described [2]. All experimental procedures reported here were approved by the veterinary authority of the Prefecture of Athens and in accordance to the National Registration (Presidential Decree 56/2013) in harmonization to the European Directive 63/2010. For the surgeries, the mice were anesthetized with isoflurane (2% deliver in oxygen) and they were subsequently mounted on a stereotactic frame. A hole was drilled over striatum (+0.7 mm anteroposterior, +1.5 mm mediolateral) and 2 μl of GL261 cells cells (2 × 10^4^) were delivered in of Dulbecco’s phosphate-buffered saline (PBS) (14040, Life Technologies Gibco) and were injected at -2.7 mm dorsoventral. After the needle was withdrawn, the incision was sutured and the mice were placed on a heated surface until fully recovered from anesthesia. The well-being of the mice was checked daily and all the operated mice reached the termination date of the study having a well-being compatible to the standards of the approved bioethical protocol. The mice were sacrificed 21 days after injection of the tumor cells. The cell viability of the GL261 cells was checked individually and for every single mouse before the operation in order to ensure that all the mice were injected with the exact same number of viable cells. Also, in order to avoid bias or other unpreceded factors interfering with the experiment, an equal number of WT and CTH KO mice was operated daily by the same operator (**Supplementary Table 2**). At termination, the mice were intracardially perfused with 4% PFA and the brains were harvested and stored in 4% paraformaldehyde solution (PFA) for 1 day. The following day the PFA was replaced by PBS with 0.1% sodium azide and the brains were stored in this solution at 4℃ until the time of the stereology and immunohistochemistry analysis.

**Stereology analysis**

Each mouse brain that had been previously perfused with 4% PFA and stored in PBS+0.1% sodium azide at 4℃, was pre-weighted, the cerebellum was then removed and then the rest of the brain was cut on 5 equal depth coronal slices with razor blades. The brain slices from each mouse were placed in two cassettes and oriented in an isotropic way ensuring that all brain areas will have an equal probability of being quantified during the stereologic analysis. After paraffin embedding, thin tissue slices were cut (4 μm) and stained with either haematoxylin and eosin (H&E) or with an antibody against the endothelial cell marker CD34 (see detailed protocol on the immunohistochemistry section below). The stained slides were digitized with a digital slide scanner. Systematic, uniform, random sampling of the regions of interest on the whole-slide images was performed. Images for the calculation of the tumor volume and the % of volume area were based on the H&E staining and were done at 10× magnification (sampling fraction 50%) using point counting. Measurements of tumoral microvessels were based on the CD34 staining and performed at 20× magnification (sampling fraction 40%). The obtained microscope images were scanned and finally analyzed by using the Visiopharm stereology software (https://visiopharm.com/visiopharm-digital-image-analysis-software-features/stereology/). The tissue slicing and counting was performed in a blinded fashion throughout the whole process.

The % of tumor area was calculated as follows: [sum of the countings in the tumor/ sum of the total countings]×100. The tumor volume was based on the estimation that the brain density is 1 mm^3^/mg tissue and calculated as follows: tumor volume (mm^3^)=total brain weight (mg)x% of tumor area. Estimations of tumor microvessel density [D(cap) in μm/μm^3^] were made on the basis of the assumption that the structures of interest are much longer than their diameter and on that the density of the brain tissue is 1 mm3/mg of tissue. The exact equation used for calculating the tumor microvessel density is the following: D(cap) = 2×Ncap total/53.503,83 μm^3^×Nref, where Ncap is the number of capillary profiles per counting area and Nref is the number of reference points within the tumor area.

**Immunohistochemistry (IHC) analysis**

The paraffin-embedded brain mouse slices used for the stereologic analysis, were also used for immunohistochemistry semi-quantitative analysis in a blinded fashion. In particular, 5 coronal slices representing randomly the whole mouse brain were stained for different primary and secondary antibodies as explained below. The primary antibody dilutions were 1/250 for CD34, 1/500 for SOX2 and 1/1000 for MAC-2. At least 5 non-overlapping, randomly positioned photos (10× magnification), covering most of the tumoral area were taken on the microscope. The intensity of DAB signal was blindly analyzed on Image J by using the ‘color deconvulation method’ as explained here https://biii.eu/colour-deconvolution .

**RNA extraction, cDNA synthesis and Real Time PCR**

Total cellular RNA was extracted by using the NucleoSpin RNA Plus kit (Macherey-Nagel GmbH & Co. KG, Duren, Germany) according to manufacturer’s instructions. The cDNA synthesis was performed by using the iScriptTM cDNA Synthesis Kit (Bio-Rad, Hercules, CA, USA) according to the manufacturer’s instructions. Real-Time PCR was performed using the created cDNA in duplicates by using a SYBR green based kit (KAPA SYBR, FAST qPCR Kit/Kapa Biosystems, Wilmington, MA, US) according to manufacturer’s instructions. The housekeeping genes was GAPDH for both mouse and human. The relative mRNA expression was calculated based on the 2^-ΔΔCt^ method based on the mRNA expression of the gene of interest and the housekeeping gene, the mRNA expression under ‘control’ conditions was set to 1 and the mRNA expression under any other experimental condition was expressed as fold changes compared to ‘control’. The detailed primer sequences are given in the **Table 1** below.

**Table 1: Primer Sequences**

| **Primer type** | **Primer Sequence (5’🡪3’)** |
| --- | --- |
| Mouse-NOX4-Fwd | TTTGTTGAAGTATCAGACAAAT |
| Mouse-NOX4-Rev | TCCAGAAATCCAAATCCAGGT |
| Human-NOX4-Fwd | GGCTGCTGAAGTATCAAACTAAT |
| Human-NOX4-Rev | TCCAGAAATCCAAAGCCAAGT |
| Mouse-CTH-Fwd | GCACCAACAGGTACTTCAGGA |
| Mouse-CTH-Rev | AACGAAGCCGACCACTTGT |
| Mouse-GAPDH-Fwd | TGTGTCCGTCGTGGATCTGA |
| Mouse-GAPDH-Rev | CCTGCTTCACCACCTTCTTGA |
| Human-CTH-Fwd | CCTGGGCTGCCCTCTCATCCA |
| Human-CTH-Rev | TGCCGGAAGCTCAGCAAGGC |
| Human-PROM-1-Fwd | ACCCAACATCATCCCTGTTCTT |
| Human-PROM-1-Rev | AGCTCTTCAAGGTGCTGTTCATG |
| Human-SOX2-Fwd | TGCGAGCGCTGCACAT |
| Human-SOX2-Rev | TCATGAGCGTCTTGGTTTTCC |
| Human-GAPDH-Fwd | GGAGTCAACGGATTTGGTCGTA |
| Human-GAPDH-Rev | GGCAACAATATCCACTTTACCA |
| Human-SOX4-Fwd | CGCGTGATGAAGACAGAAGGCTCCG |
| Human-SOX4-Rev | AAACGGGAATTCGCCTGCGTGG |
| Human-NES-Fwd | AGCCCTGACCACTCCAGTTTAG |
| Human-NES-Rev | CCCTCTATGGCTGTTTCTTTCTCT |

**NOX4 activity/H_2_O_2_ measurements**

NOX4 activity was performed as previously described [3] based on an Amplex Red fluorescence method with some slight modifications for cell measurements. The fluorescence was measured at 37℃ in a black 96-well plate (fluronunc black, Nunc, ThermoFisher Scientific, Cat No. 237108) after the addition of NADPH (100 μΜ, Sigma N5130), AmpliFlu Red (100 μM, Sigma 90101) and horse radish peroxidase (1 U/mL, Sigma, Cat No. P-8250-5KU). In all cases, appropriate blank samples without any cells, NADPH or the fluorescent probe were used for validating the reliability of the method. The final RFU (relative fluorescence unit) signal was normalized according to the number of cells/well.

NOX4 activity was performed as previously described based on an Amplex Red fluorescence method with some slight modifications for cell measurements. The fluorescence was measured at 37℃ in a black 96-well plate (fluronunc black, Nunc, ThermoFisher Scientific, Cat No. 237108) after the addition of NADPH (100 μΜ, Sigma N5130), AmpliFlu Red (100 μM, Sigma 90101) and horse radish peroxidase (1 U/mL, Sigma, Cat No. P-8250-5KU). In all cases, appropriate blank samples without any cells, NADPH or the fluorescent probe were used for validating the reliability of the method. The final RFU (relative fluorescence unit) signal was normalized according to the number of cells/well.

**Supplemental Information on Mouse Surgeries**

Eight out of eight WT mice developed GBM tumors 21 days (termination date based on previous well-established protocols i.e. Sci Signal. 2015 Dec 8;8(406):ra125. doi: 10.1126/scisignal.aaa1690) after the injection of 2x10^4^ mouse GBM cells whereas for the *CTH* KO the respective number was three out of eight (see **Table 2** below). We are confident that this finding reflects the role of CTH expression in the tumor host and it is not because of any technical mistake sine the surgeries were performed in a such way ensuring that: i) the same number of WT and *CTH* KO were operated in an alternated way on every operation day, ii) the same number of alive GBM cells (cell viability was measured just before the intracranial injection) were injected on both WT and *CTH* KO, iii) on every operation date the WT and *CTH* KO were injected with GBM cells derived from the same vial (and therefore the same cell batch and passage), iv) the three *CTH* KO mice that developed tumors, were operated in three different operation dates and therefore there was no time factor affecting the success rate of the operations, v) importantly, on every operational date all the WT mice developed large and measurable tumors, vi) all the operated mice were of exactly the same age and gender.

**Table 2: Information on mouse surgeries**

| Surgery date |  | Genotype | Number of injected alive GBM cells | Coordinates (site of injection) | Visible (by IHC) tumor developed | Gender | Age (weeks) |
| --- | --- | --- | --- | --- | --- | --- | --- |
| 17/12/2020 |  | WT | 20000 | M/L: 29,7 - A/P: 58,8 - D/V: 57,5 | YES | F | 8 |
| 18/12/2020 |  | WT | 20000 | M/L: 24,6 - A/P: 59,6 - D/V: 57,7 | YES | F | 8 |
| 18/12/2020 |  | WT | 20000 | M/L: 24,4 - A/P: 56,9 - D/V: 58,1 | YES | F | 8 |
| 21/12/2020 |  | WT | 20000 | M/L: 21,6 - A/P: 59,7 - D/V: 57,5 | YES | F | 8 |
| 21/12/2020 |  | WT | 20000 | M/L: 26 - A/P: 55,8 - D/V: 28,4 | YES | F | 8 |
| 21/12/2020 |  | WT | 20000 | M/L: 23,4 - A/P: 58 - D/V: 58,6 | YES | F | 8 |
| 22/12/2020 |  | WT | 20000 | M/L: 22,9 - A/P: 59,1 - D/V: 58,1 | YES | F | 8 |
| 22/12/2020 |  | WT | 20000 | M/L: 23 - A/P: 59,1 - D/V: 58,4 | YES | F | 8 |
| 17/12/2020 |  | *CTH* KO | 20000 | M/L: 24,1 - A/P: 59,4 - D/V: 56,9 | YES | F | 8 |
| 18/12/2020 |  | *CTH* KO | 20000 | M/L: 24,4 - A/P: 58,7 - D/V 57,6 | NO | F | 8 |
| 18/12/2020 |  | *CTH* KO | 20000 | M/L: 22,8 - A/P: 59 - D/V: 58,8 | YES | F | 8 |
| 21/12/2020 |  | *CTH* KO | 20000 | M/L: 25,1 - A/P: 59,5 - D/V: 57,8 | NO | F | 8 |
| 21/12/2020 |  | *CTH* KO | 20000 | M/L: 23 - A/P: 59,1 - D/V: 57,4 | YES | F | 8 |
| 21/12/2020 |  | *CTH* KO | 20000 | M/L: 23,8 - A/P: 57,5 - D/V: 58,3 | NO | F | 8 |
| 22/12/2020 |  | *CTH* KO | 20000 | M/L: 22,7 - A/P: 59,1 - D/V: 58,3 | NO | F | 8 |
| 22/12/2020 |  | *CTH* KO | 20000 | M/L: 23,2 - A/P: 58,5 - D/V: 58,1 | NO | F | 8 |

**Comparison of evaluation of tumor area and tumor volume by blinded microscopy and by blinded stereology**

In order to fully confirm that the genetic ablation of CTH in the tumor host attenuates GBM formation:

We used the **same coronal slices** that were used for the stereology analysis but the percent of tumor area and tumor volume was measured by **blinded microscopy** based on the H&E staining. The tumor area was measured on Image J by comparing the area of the tumor area vs healthy brain in non-overlapping photos at 10 x magnification. The tumor volume was measured with the same principle as for stereology: tumor volume (mm^3^)=tumor area X brain weight (mg)/100 assuming again that that the density of the brain tissue is 1 mm^3^/mg. All the brain areas from all the coronal slices were photographed. As seen in **Supplemental Figure 2A and 2B**, the % of tumor area and the tumor volume (mm^3^) is slightly overestimated with the blinded microscopy and Image J analysis compared to the stereology counting (see **Figure 1E**). However, the conclusion remains the same, that the *CTH* KO have a significantly lower % of tumor area. In **Supplemental Figure 2A**, we included in the analysis all the WT and *CTH* KO mice that underwent a surgery and were injected the mouse GBM cells irrespective of if they developed at the end tumors or not (N=8/ group). In **Supplemental Figure 2C and 2D**, the same analysis by blinded microscopy was performed but only in the mice bearing visible tumors at termination/3 weeks after the injection of GBM cells (N=8 for WT, N=3 for *CTH* KO). Again, these 3 *CTH* KO are characterized by a significantly lower % of tumor area and tumor volume compared to WT.

Again, our results indicate that there is a slight overestimation of the % of tumor area and tumor volume (mm^3^) by blinded microscopy compared to stereology (comparison of **Supplemental Figures 2C, D vs Figure 1F**). All the mean±SEM values for comparing the blinded microscopy and blinded stereology are given in the **Table 3** below:

**Table 3: Comparison of blinded microscopy and stereology for tumor volume and area analysis**

|  |  | **Microscopy** | **Stereology** |
| --- | --- | --- | --- |
| WT (8 out of 8 operated and developed tumors) | **% tumor area** | 16.26±3.673 | 11.29±1.812 |
|  | **Tumor volume (mm^3^)** | 92.47±23.69 | 63.70±12.03 |
| *CTH* KO (8 operated) | **% tumor area** | 2.72±1.293 | 1.961±1.077 |
|  | **Tumor volume (mm^3^)** | 11.03±6.343 | 9.35±5.215 |
| *CTH* KO (3 out of the 8 operated that developed tumors) | **% tumor area** | 6.059±3.519 | 5.23±1.511 |
|  | **Tumor volume (mm^3^)** | 29.41±10.26 | 24.93±7.714 |

**Supplemental information on the Results**

**CTH is expressed in various GBM cell lines.**

CTH expression was confirmed by Real Time PCR on the mouse GBM cell line GL261 used for the in vivo surgeries and in two different human GBM lines (**Supplemental Figure 1**).

**The attenuated GBM formation on *CTH* KO mice is confirmed by blinded microscopy.**

As a complementary analysis to the golden standard stereological analysis, we also performed blinded microscopy analysis on the same slides and calculated the % tumor area and the tumor volume (mm^3^). The analysis was performed on all operated mice (**Supplemental Figure S2A-B**) and on all tumor bearing mice (**Supplemental Figure S2C-D**). In all cases the *CTH* KO are characterized by significantly lower % tumor area and tumor volume compared to the WT.

**CTH expression is not positively correlated with the GSC markers NES, PROM1 and CD44 based on bioinformatic analysis.**

Bioinformatic analysis on data derived from the cBioPortal for Cancer Genomics suggest that *CTH* mRNA expression is not positively correlated with the GSC markers *NES* (**Supplemental Figure 3A**), *PROM1* (**Supplemental Figure 3B**) or *CD44* (**Supplemental Figure 3C**).

**CTH expression is not positively correlated with the EC marker CLDN5 based on bioinformatic analysis.**

Bioinformatic analysis on data derived from the cBioPortal for Cancer Genomics suggest that *CTH* mRNA expression is not positively correlated with the EC marker *CLDN5* (**Supplemental Figure 3D**).

**Higher CTH mRNA expression in patients with recurrent gliomas tends to be associated with lower survival probability.**

Higher *CTH* mRNA expression is associated with a clear trend (P-value: 0.056) for lower survival probability in recurrent gliomas of all grades as indicated by bioinformatic analysis on data derived from the CGGA atlas (**Supplemental Figure 3E**).

**SOX2 expression on various GBM lines is not changed upon CTH pharmacological inhibition.**

SOX2 protein (**Supplemental Figure 4A**) or mRNA (**Supplemental Figure 4B-C**) expression is not altered after administration of PAG for 24h compared to ‘Control (0.1% DMSO) treated cells on mouse (**A**) or human GBM cells (**B-C**).

**The brain permeable pharmacological inhibitor of CTH, PAG, is not a cytotoxic drug on various GBM cell lines.**

PAG does not have any major direct cytotoxic effects on different human and mouse GBM cells since even very high PAG concentrations (at the mM range) are not critically cytotoxic (the % of cell viability was not lower than 45-50% with any of the tested PAG concentrations) (**Supplemental Figure 5**).

**The H_2_S donor, NaSH is not a cytotoxic drug on mouse GBM cells.**

Application of various concentrations (0.07-1.25 mM) of the H_2_S donor, NaSH directly on the mouse GBM cells GL261 does not have any major cytotoxic effect, except from a mild cytotoxic effect at 0.3 mM (**Supplemental Figure 6A**).

**The H_2_S donor, NaSH has a mild anti-proliferative effect on mouse GBM cells.**

Treatment of the mouse GBM cells GL261 with two more physiologically and/or pharmacologically relevant concentrations (0.15, 0.3 mM) of NaSH led to a mild but significant reduction of cell proliferation (**Supplemental Figure 6B**).

**Pharmacological CTH inhibition attenuates the TGF-β induced cell migration and ROS production (NOX4 activity) on mouse GBM cells.**

TGF-β triggers higher GL261 cell migration (**Supplemental Figure 7A**) and ROS production (**Supplemental Figure 7B**), an effect attenuated significantly by PAG.

**Supplemental Figures and Figure Captions**

**Supplemental Figure 1: CTH expression in different GBM cell lines as confirmed by Real Time PCR.** CTH expression was confirmed in the mouse GBM line (GL261) used for the *in vivo* GBM model and two different human lines (U3031MG, U3017MG). The graph visualizes the average 2^-ΔCt^ values for each cell line and the error bars indicate the SEM. N=4 independent experiments/group for GL261, N=2 average of 2 biological replicates/group for U3031MG and U3017MG cells.

**Supplemental Figure 2: Comparison of evaluation of tumor area and tumor volume by blinded microscopy. A.** % of the tumor area in all operated mice as calculated by blinded microscopy, N=8/group for WT and N=8/group for *CTH* KO mice, **B.** tumor volume (mm^3^) as calculated by blinded microscopy in all operated mice, N=8 for WT, N=8 for *CTH* KO. **C.** % of tumor area on the tumor bearing mice only at termination N=8/group for WT and N=3 for *CTH* KO as calculated by blinded microscopy, **D.** tumor volume (mm^3^) on the tumor bearing mice only at termination N=8/group for WT and N=3 for *CTH* KO as calculated by blinded microscopy. *p < 0.05; **p < 0.01 , Unpaired Student’s t-test.

**Supplemental Figure 3:** **CTH expression and overall survival in recurrent glioma and correlation with other GSC and EC markers. A-C:** Non-significant correlations between CTH mRNA expression and the mRNA expression of the GSC markers NES **(A),** PROM1**(B)** and CD44 (C). D**.** Non-significant correlation between CTH mRNA expression and the EC marker CLDN5**. E.** GBM patients with higher tumor CTH mRNA expression levels have a clear trend for lower survival probability in all grades of recurrent gliomas compared to GBM patients with lower CTH expression (CGGA atlas). The individual patient correlation expression data (panels **A-D**) are normalized from Illumina HiSeq_RNASeqV2 data set (cBioPortal for Cancer Genomics), blue dots: patients with non-mutated CTH or both proteins of interest, yellow dots: patients with mutated CTH, red dots: patients with both proteins of interest mutated, white dots: patients not profiled for mutation. R^2^ and statistical significance values after Pearson correlation tests are shown.

**Supplemetal Figure S4: SOX2 expression upon CTH inhibition in various GBM cell lines.** **A**. SOX2 protein expression in mouse GL261 cells on Control (0.1%DMSO, 24h) or PAG (600 μΜ, 24h) treated cells, N=6/group. **B-C:** SOX2 mRNA expression in human U3017MG **(B)** and U3031MG **(C)** cells on Control (0.1%DMSO, 24h) or PAG (600 μΜ, 24h) treated cells. N=2 biological replicates/group. Unpaired Student’s t-test

**Supplemental Figure 5: Cell Viability of different GBM cell lines with increasing concentrations of PAG.** Cell Viability (%) with increasing concentrations of PAG (24h) in U3031MG (**A**), U3017MG (**B**), U3034MG(**C**) and GL261 (**D**) cells. N=3/group for U3031MG, U3017MG and U3034MG cells, N=6/group for the GL261 cells. *p < 0.05 vs ’Control/Vehicle treated cells’, Unpaired Student’s t-test

**Supplemental Figure 6**: **Effect of the H_2_S donor, NaSH, on cell proliferation and viability of mouse glioblastoma cells.** **Α.** Cell Viability (%) with increasing concentrations of NaSH (24h) in GL261 mouse glioblastoma cells. **B.** % Cell proliferation of two more physiologically and/or pharmacologically relevant concentrations (0.15 and 0.3 mM) of NaSH. N=6/group for for both A and B. *p < 0.05 vs ’Control treated cells’, Unpaired Student’s t-test

**Supplemental Figure 7. CTH inhibition attenuates the TGF-β induced NOX4 activity and cell migration. A-B.** **A**. Wound closure over time (% of closure area of 0 vs 6 h) for Control (0.1% DMSO+0.1% BSA in 4mM HCl/solvent for TGF-β), TGF-β (TGF-β 5ng/ml+0.1%DMSO, 6h) and TGF-β (5ng/ml)+PAG (600 μΜ, 6 h) treated cells. N=3/group. **Β**. NADPH oxidase activity under the same experimental conditions but with duration of the treatment for 48h. N=8-12/group. Comparisons between 3 groups over time were performed by two-way ANOVA followed by Bonferroni’s multiple comparison test (**A**). Comparisons between 3 groups were performed by ordinary one-way ANOVA followed by Bonferroni’s multiple comparison test (**B**). *p < 0.05, ***p<0.001 vs the indicated group.

REFERENCES

1. Cano-Galiano, A., et al., *Cystathionine-gamma-lyase drives antioxidant defense in cysteine-restricted IDH1-mutant astrocytomas.* Neurooncol Adv, 2021. **3**(1): p. vdab057.

2. Yang, G., et al., *H2S as a physiologic vasorelaxant: hypertension in mice with deletion of cystathionine gamma-lyase.* Science, 2008. **322**(5901): p. 587-90.

3. Peleli, M., et al., *Renal denervation attenuates hypertension and renal dysfunction in a model of cardiovascular and renal disease, which is associated with reduced NADPH and xanthine oxidase activity.* Redox Biol, 2017. **13**: p. 522-527.
